# Data-driven prediction of thin-film metallic glass forming ability *via* Bayesian classification and experimental verification in the Cu–Zr–Al system

**DOI:** 10.1039/d6ra07674b

**Published:** 2026-09-11

**Authors:** Xuliang Luo, Tero Mäkinen, Wenyi Huo, Silvia Bonfanti, Zhehao Chen, Kenichiro Mizohata, Kostas Sarakinos, Mikko Alava

**Affiliations:** a Department of Applied Physics, Aalto University PO Box 11000 00076 Aalto Espoo Finland tero.j.makinen@aalto.fi mikko.alava@aalto.fi; b NOMATEN Centre of Excellence, National Center for Nuclear Research 05-400 Otwock Poland; c Center for Complexity and Biosystems, Department of Physics ‘Aldo Pontremoli’, University of Milan Milano Italy; d Department of Physics, University of Helsinki PO Box 43 FI-00014 Helsinki Finland; e KTH Royal Institute of Technology, Department of Physics Roslagstullsbacken 21 114 21 Stockholm Sweden

## Abstract

Identifying alloy compositions suitable for metallic glass formation from the vast and multi-dimensional space of elemental combinations remains a significant challenge. In the present study, we develop—using literature data—a Bayesian machine learning model based on Gaussian process classification for predicting the glass-forming ability (GFA) of metal-alloy compositions. The model incorporates a wide variety of descriptors computed based on the alloy compositions and physical properties of the constituent elements. With the optimal descriptor set the model achieves a prediction accuracy of 87% on an independent test set. The model shows predictive power that encompasses multiple alloy systems, and in this work we demonstrate this power by focusing on three ternary systems for which we compute GFA for multiple compositions that are not in the test set. Additionally, archetypal Cu–Zr–Al thin-film alloys of selected compositions are synthesized using magnetron sputtering and compared with the model predictions. By characterizing the as-deposited sample structures and their evolution under heat treatment, we verify that Cu–Zr–Al system tends to have better GFA when the Al content is less than 20%. The overall results presented herein show that the model captures the composition-GFA relationships well, and further provides valuable guidance for the design of a wide range of amorphous alloys.

## Abstract 2

A probabilistic machine learning framework predicts thin-film metallic-glass formation from composition-derived descriptors and is experimentally validated in Cu-Zr-Al system.

## Graphical abstract

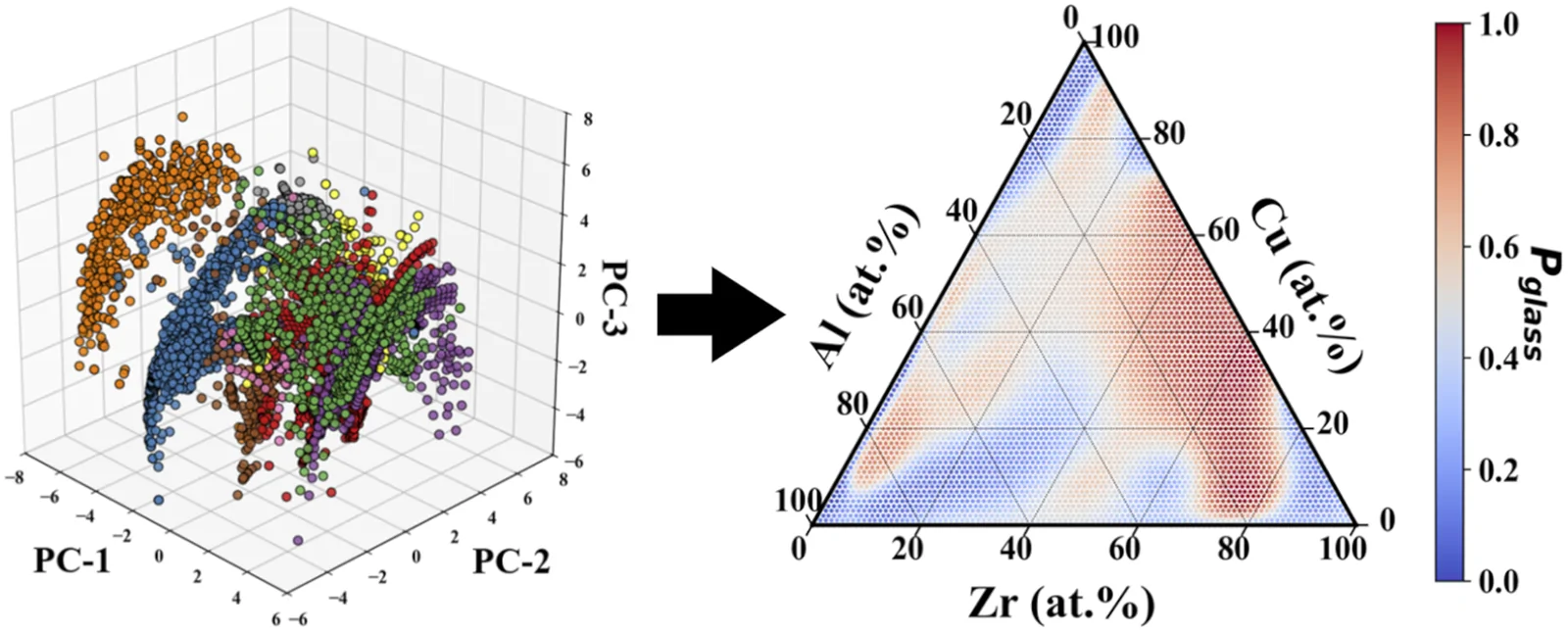

## Introduction

Metallic glasses (MGs) are a class of non-crystalline alloys characterized by the absence of long-range order while often exhibiting short-range ordering.^1–3^ This atomic arrangement yields materials with a unique combination of physical properties, including high specific strength, large elastic limits,^4,5^ good corrosion and wear resistance,^6–8^ and—in certain alloy systems—excellent soft magnetic performance.^9,10^ These properties make MGs attractive for a range of structural and functional applications.

The formation of an amorphous structure in metal alloys is a non-equilibrium process, in which the liquid is rapidly cooled to bypass crystallization. The resulting amorphous solid is metastable, with higher free energy than the corresponding crystalline phase.^11^ A key metric in this context is the glass-forming ability (GFA), which describes the ease with which a metallic alloy can form a glass. GFA is commonly evaluated by the critical cooling rate required to suppress crystallization or by the maximum casting diameter achievable under a given cooling rate.^12–17^ While in principle nearly any alloy can form a glass if cooled rapidly enough,^18,19^ practical limitations on cooling rates impose stringent constraints on achievable compositions, especially in bulk form.

Conventional approaches for fabricating bulk metallic glasses (BMGs)—such as copper mold casting, suction casting, spark plasma sintering and other methods—are typically limited to cooling rates on the order of 10^2^ to 10^4^ K s^−1^,^20,21^ which severely restricts the range of compositions that can be vitrified. In contrast, vapor-phase techniques (including magnetron sputtering) enable atom-by-atom deposition of thin-films, achieving effective cooling rates up to 10^13^–10^14^ K s^−1^.^22,23^ These extreme quenching rates allow access to a significantly broader compositional space and make it possible to form glassy structures in alloys that would crystallize under slower cooling conditions. However, even under these very-far-from-equilibrium conditions, not all compositions yield amorphous films,^24,25^ underscoring the continued importance of alloy composition in determining glass-forming ability.

To efficiently explore the vast compositional design space, high-throughput synthesis techniques—such as combinatorial magnetron sputtering—have been developed to rapidly screen thin-film metallic glass (TFMG) forming alloys^26–34^ and to establish databases. However, even such methods face challenges in covering the vast number of possible compositions. For example, considering only combinations of common glass-forming elements, the number of possible ternary, quaternary, and quinary alloys already exceeds several millions. In this context, data-driven methods and machine learning (ML) offer powerful tools for prioritizing candidate compositions and accelerating discovery.^35–40^

In the present study, we develop and experimentally validate a machine learning framework to predict glass formation in thin-film alloys based on their composition and derived descriptors. Using a curated dataset of 6835 TFMG compositions (binary and ternary based on a pool of 51 metal elements) from the Landolt–Börnstein (LB) handbook^44^ and prior literature,^45^ we compute composition-based descriptors capturing geometric, electronic, thermodynamic, and mechanical characteristics.^46–48^ Principal component analysis (PCA) is used for dimensionality reduction,^49^ followed by Bayesian classification using Gaussian process classification (GPC) to distinguish glass-forming from non-glass-forming compositions. Our test set consists of two parts, one from the LB handbook TFMG compositions, but independent of the training set, while the other part is from experimental data containing quaternary and quinary compositions reported in the literature,^35^ which are used to evaluate the model accuracy.

The model achieves an accuracy of ∼87%, and we then deploy it to predict the composition-structure map of archetypal metallic glass systems. Our analysis shows that predicting glass formation under thin-film deposition conditions involves a complex mixture of compositional disorder, thermodynamic properties, electronic structure properties, atomic radii, and other bonding parameters. To validate the predictive capacity of the model, we experimentally synthesize Cu–Zr–Al alloy films by magnetron sputtering and compare their structure to the model predictions. The results confirm the compositional dependence of GFA for Cu–Zr–Al thin alloy films and demonstrate the effectiveness of our combined computational–experimental approach for guiding TFMG and amorphous alloy discovery.

## Methods

### Data collection

**Fig. 1:**
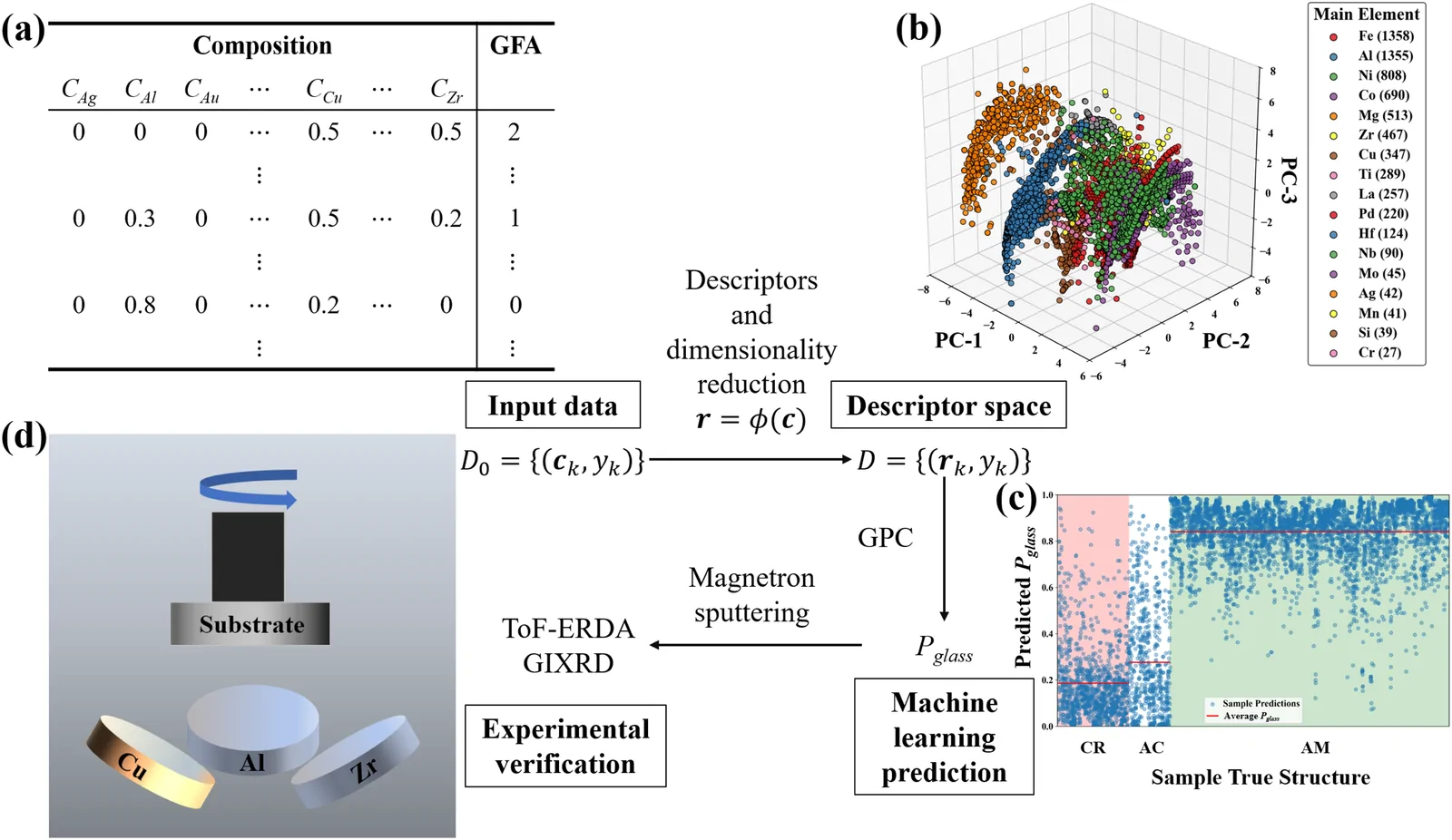
The workflow of our prediction process. (a) Input data *D*_0_ consisting of compositions *c* and their GFA classification CR, AC and AM is collected from the literature. (b) By calculating the descriptors based on the compositions, and performing dimensionality reduction to *n* dimensions using PCA (all denoted by the function *ϕ*), gives the location *r* in the descriptor space used as the input data *D* for the GPC. Here a 3-dimensional space is illustrated and the main element of each composition highlighted by the color. (c) The GPC gives for each composition a probabilistic prediction *P*_glass_ for the GFA. (d) Thin film samples of selected compositions are synthesized and annealed post-deposition using magnetron sputtering, and characterized using time-of-flight elastic recoil detection analysis (ToF-ERDA) and grazing incidence X-ray diffractometry (GIXRD), to test the predictions.

The overall workflow for the training and prediction process is schematically illustrated in Fig. 1. In the remainder of the present section we describe the various parts of our research methodology as indicated in the corresponding sub-sections. Firstly, a dataset with GFA labels and compositions is input (Fig. 1a). The TFMG dataset which we use to train the Bayesian Classification (BC) model comes from the work published by Ward *et al.*,^45^ which was screened from the LB-handbook^44^ collection and experimentally evaluated. The dataset contains a total of 6835 binary and ternary alloys, of which the number of amorphous (AM), amorphous-crystalline (AC) and crystalline (CR) samples are 4869, 721, and 1245, respectively. The alloys are based on metallic elements that are selected from a group that primarily contains Fe, Al, Ni, Co, Mg, Zr and Cu, with additional elements shown in Fig. 1b. The TFMG dataset contains some duplicate compositions produced by multiple fabrication routes (*e.g.*, melt spinning, suction casting, magnetron sputtering, *etc.*). Simply deleting duplicated compositions or assigning a positive label whenever an AM report exists would not be appropriate for the present research purpose. The processing route is not included as a descriptor. The input data and, thereby, the model predictions should therefore be considered as reflecting the GFA under the cooling rates typically achieved by commonly used thin-film sample preparation methods, which are included as hidden variables in the data. Conflicting labels for duplicated compositions do not prevent the GPC model from being trained. Instead, the GPC accommodates these repeated observations through its probabilistic prediction, which represents a compromise between the conflicting labels. Because each observation contributes separately to the likelihood, repeated compositions effectively increase the statistical weight of that composition. For example, if a composition appears multiple times in the dataset and is reported as AM in 7 cases and non-AM in 3 cases, the empirical frequency would more likely to suggest a higher GFA for that composition. Our test set comes from 562 TFMG samples (36 binary, 367 ternary, 84 quaternary and 75 quinary alloys) independently from the training set, which are screened from part of LB handbook and the report of Garel *et al.*,^35^ including 249 AM and 313 non-AM class compositions. The phase labels were treated as three categorical classes during model training: CR, AC, and AM. In the implementation, these classes were encoded as CR = 0, AC = 1, and AM = 2, where the numerical values serve only as class identifiers and do not imply an ordinal or continuous target. The Gaussian process classifier was trained on these three classes. For the binary performance evaluation, AM was defined as the positive class, AC and CR were grouped together as the non-AM negative class. Thus, AC samples were included in the binary evaluation as non-AM samples rather than being removed or treated as intermediate positive samples.

### Descriptors

Descriptors are key factors in the training process of machine learning models. According to a large number of studies on machine learning for metallic glasses in recent years, we select 19 different elemental properties (see Table 1), and then the linear mixing1
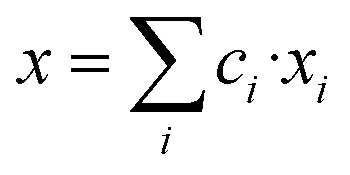
and standard deviation of properties2
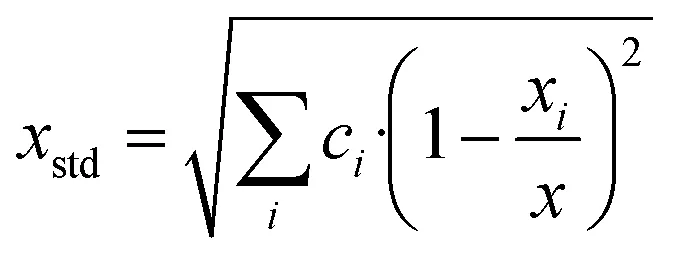
are calculated to obtain 38 elemental descriptors (EDs), where *c*_*i*_ represents the content of the element *i* in the alloy, and *x*_*i*_ is the value of its corresponding property. For EDs, *x* represents the average characteristic of the constituent elements of the alloy composition (*e.g.*, the average atomic radius), while *x*_std_ represents the characteristic differences. The EDs, PDs, and combined descriptors (CDs) are used separately to train GPC models for comparison.

**Table 1:** The properties used for calculating the elemental descriptors and physical descriptors

Elemental descriptors (EDs)	Covalent radius	Metallic radius	Density
	Valence electron concentration (VEC)	Valence	Thermal conductivity
	Miedema electronegativity	Period	Electron affinity
	Pauling electronegativity	Chemical potential	Melting temperature
	Boiling temperature	molar heat capacity	Fusion enthalpy
	Thermal expansion	Debye temperature	Vaporization enthalpy
	Wigner–Seitz electron density		
Physical descriptors (PDs)	Ideal entropy^41^	Xia's ideal entropy^42^	Mismatch entropy^3^
	Mixing entropy	Mixing enthalpy^3^	Mixing Gibbs free energy
	Lattice distortion	Mixing *P*_HS_^43^	Mixing *P*_HSS_^43^
	Standard deviation of the bulk modulus	Bulk modulus	

It should be noted here that the contribution of constituent elements to the properties of the alloys may be more complex than that captured in the EDs. Hence, based on atomic radius and considering physical quantities such as mismatch and mixing entropy, physical descriptors (PDs) are calculated through specific equations^46,48,50,51^ shown in the SI Material (SM) in Table I. In total, eleven PDs are calculated based on atomic radius, mixing entropy and bulk modulus. Table 1 lists all the descriptors we use.

### Machine learning model

Due to the large number of (possibly correlated) descriptors used, dimensionality reduction is needed before the GPC is performed. Here we do this using PCA to avoid introducing any distortions to the input data, which might be possible with nonlinear dimensionality reduction methods.^49^ Irrespective of the number of descriptors, we can use *n* principal components as the input data to train the GPC. Before performing PCA, all input data are standardized by subtracting the descriptor mean *µ*_*k*_ and dividing by the standard deviation *σ*_*k*_

3 *z*_*k*_ = (*x*_*k*_ − *µ*_*k*_)/*σ*_*k*_

to ensure that the final mean of each descriptor *z*_*k*_ is 0 and the standard deviation is 1. Fig. 1b shows input data distribution in 3D-PCA space, the main element refers to the element present in the greatest proportion in the alloy composition.

The core of the machine learning model we use is BC based on the probabilistic Gaussian process classification (GPC), as implemented in the scikit-learn library.^52^ Gaussian processes^53,54^ are usually used for Gaussian process regression (GPR), but in classification tasks we want to predict the class probability rather than the continuous value. This is done by first defining a nuisance function *f*, which is a Gaussian process4
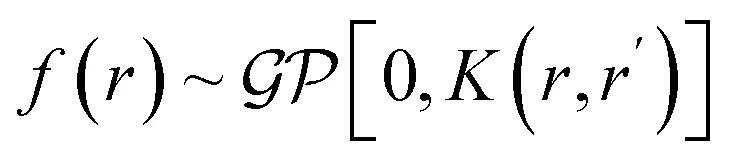
where *r* = [*r*_1_, *r*_2_, …, *r*_*n*_] is the input feature vector after PCA dimension reduction, and where *n* is the number of principal components used. The *K*(*r*, *r*′) is the kernel function, and the anisotropic radial basis function (RBF) kernel5
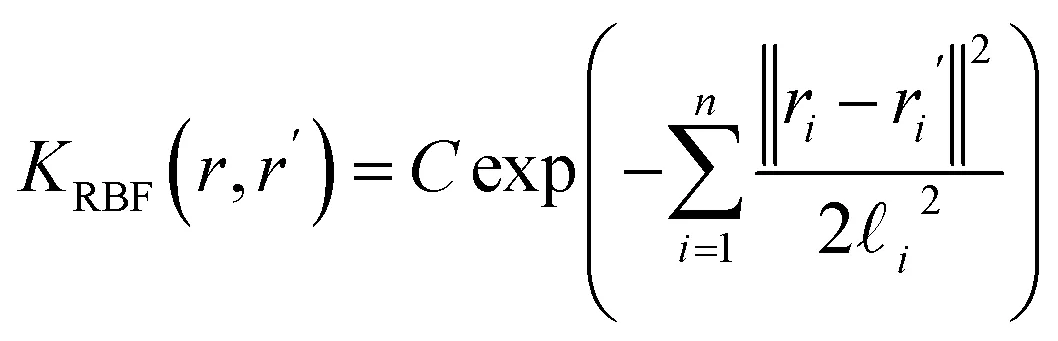
where 
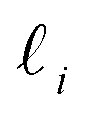
 is a length scale hyperparameter (corresponding to the *i*th principal component) that controls the extent of the correlations in the predictions, *i.e.* the smoothness of *f*. The value of 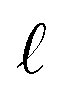 is automatically optimized within the range (10^−2^, 10^2^) during the training process. *C* is a constant representing the global amplitude of the kernel function, and ‖*r* − *r*′‖^2^ represents the Euclidean distance between *r* and *r*′ in the PCA reduced space. As we have three classes (AM, AC, and CR), the GPC uses a one-vs-rest strategy, and we have separate length scales for each class.

For each point in the input space, GPR gives a Gaussian distribution of the values of the nuisance function, characterized by the mean and the standard deviation. The kernel then measures the similarity of the distribution at two different points. To yield the GPC prediction, this nuisance function is transformed by a logit-transform to give values between 0 and 1. Then as shown in Fig. 1c, the posterior mean is computed using the laplace approximation to yield a single valued prediction for the probability of belonging to a class, *i.e.*, the probability that the composition is glass *P*_glass_.

After the model training is completed, the probability of each composition corresponding to three classes is output, and experimental verification (Fig. 1d) can be performed by synthesizing the thin-film samples. All computations are performed on the Triton high-performance computing cluster at Aalto University. Training a single GPC model with nine retained principal components typically required approximately 20 hours under our computational settings.

### Experimental methods

#### Magnetron sputtering

To verify the model predictions, we study the microstructure of Cu–Zr–Al alloys synthesized in the form of thin-films by magnetron sputtering. Thin Cu–Zr–Al films with various compositions are deposited by magnetron co-sputtering on Si(001) substrates covered with a ∼300 nm thermally grown silicon dioxide layer. The substrates have dimensions 10 × 10 × 0.5 mm^3^ and are cleaned in isopropanol in an ultrasonic bath for 10 min prior to the deposition. Experiments are performed in an ultra-high vacuum deposition system (base pressure ∼10^−6^ Pa), equipped with a load-lock chamber and three magnetron sources. The target-to-substrate distance is 190 mm and the target surface vector forms an angle of 45° with respect to the substrate normal vector. The substrate holder is rotated at a constant speed of 3 rpm to obtain uniform deposition over the substrate area. Ar (99.99% purity) is used as the sputtering gas, and the discharge pressure is maintained at 0.25 Pa during deposition. Cu, Zr, and Al targets (purity 99.995%; diameter 50.8 mm) are used for the deposition process. Direct current (DC) power is applied to the Zr target, while the Cu and Al targets are supplied with radio-frequency (RF) power from two separate generators. DC and RF powers are adjusted in the ranges 0–120 W (Cu), 0–90 W (Zr), and 0–140 W (Al) to control the film composition (see Table 2), while maintaining a deposition rate of ∼4 × 10^14^ atoms cm^−2^ s^−1^. The deposition time for the samples is approximately 800–1200 s, resulting in a final film thickness of around 100 nm. No intentional heating is applied on the substrate during deposition.

**Table 2:** Magnetron sputtering parameters and sample composition for the synthesized alloys. The uncertainty of all elemental concentrations measured by TOF-ERDA is approximately ± 0.5 at%

Sample	Composition (at%)	Cu power (W)	Zr power (W)	Al power (W)
T1	Cu_15_Zr_59_Al_26_	11	88	27
T2	Cu_12_Zr_26_Al_62_	15	65	135
T3	Cu_14_Zr_31_Al_55_	12	45	70
T4	Cu_9_Zr_35_Al_56_	11	63	89
T5	Cu_16_Zr_22_Al_62_	15	36	89
T6	Cu_25_Zr_59_Al_16_	18	86	17
T7	Cu_22_Zr_66_Al_12_	15	85	11
T8	Cu_21_Zr_59_Al_20_	16	89	21
T9	Cu_20_Zr_64_Al_16_	14	83	15
T10	Cu_13_Zr_67_Al_20_	10	88	16
T11	Cu_55_Zr_0_Al_45_	61	0	131
T12	Cu_69_Zr_3_Al_28_	118	15	131
T13	Cu_13_Zr_81_Al_6_	7	89	7

#### X-ray reflectometry

Initial values of power applied to the three sputtering cathodes are selected based on X-ray reflectometry (XRR) measurements, which are used to determine the film thickness and mass density and hence the deposition rate from each target (see SM, SI Table II). XRR measurements are performed in a home-built diffractometer comprising a Huber four-circle goniometer, a Seifert ID3003 X-ray tube (Panalytical Cu anode Fine focus, type PW2213/20), a Huber Germanium (111 reflection) monochromator, and a Minipix D05-W0051 detector. The detector acts as a receiving slit, whereby a single column of pixels corresponds to a divergence angle of in the scale. Measurements are performed in the 2*θ* range of 0–3°. XRR curves are analyzed using “GenX”^55^ software package.

#### Time-of-flight elastic recoil detection analysis

Film composition is determined by time-of-flight elastic recoil detection analysis (ToF-ERDA) in the EGP-10-II 5 MV tandem accelerator of the Helsinki Accelerator Laboratory.^56^ Measurements are performed using a ^127^I ion beam, with the detector positioned at an angle of 40° with respect to the direction of the incident beam, while the beam incident direction forms an angle of 20° with respect to the sample surface. Compositions are calculated using stopping forces obtained from the SRIM freeware,^57^ the measurement geometry, and Rutherford elastic recoil cross sections for the detected elements. Table 2 lists the synthesized thin-film samples, along with the power applied on the three sputtering cathodes and the corresponding composition determined by ToF-ERDA.

#### Grazing incidence X-ray diffractometry

The microstructure and phase composition of the alloys is determined using Grazing Incidence X-ray Diffractometry (GIXRD). GIXRD measurements are performed using a Rigaku SmartLab X-ray diffractometer equipped with Cu Kα radiation operated at 45 kV and 40 mA. The grazing incident angle is set to 1°, and the 2*θ* range is from 20° to 70° with a 5° min^−1^ scanning rate.

#### Thermal annealing

Temperature indicators such as *T*_g_ (glass transition temperature) and *T*_*x*_ (crystallization temperature) are considered glass stability parameters and are related to the GFA.^58,59^ To evaluate the stability of our alloys, as-deposited thin-film samples are subjected to thermal annealing. The samples are annealed in a tube furnace under ultra-high vacuum (10^−6^ Pa). Annealing is initiated at a temperature of 723 K after which the temperature is increased at increments of 30 K up to a value of 813 K. Each annealing step has a duration of 1 h.^60^ The microstructure of annealed samples is studied by GIXRD.

#### Focused ion beam and high-resolution transmission electron microscopy

A JIB-4700F Focused Ion Beam (FIB) System is used to prepare cross-sectional lamellae for Transmission Electron Microscopy (TEM) analysis. The standard TEM lift-out procedure^61^ is performed in the surface region. The region of interest is first covered with protective layers of platinum (Pt) and carbon (C) to prevent further ion-induced damage prior to lamella extraction. Subsequently, the lamella is thinned to approximately 100 nm by successive ion milling and polishing steps. Coarse milling is carried out using a 30 keV gallium (Ga) ion beam with a current of 500 pA. This is followed by a final polishing step using a 5 keV Ga ion beam with a current of 30 pA to remove the additional damage generated during the thinning process.

A High-Resolution Transmission Electron Microscope (HRTEM; JEOL JEM-2200FS aberration-corrected transmission electron microscope, spatial resolution of 0.1 nm, operated at an accelerating voltage of 200 kV) is employed to investigate the general microstructure and crystallinity of the films. In addition, Fast Fourier transform (FFT) analysis of the HRTEM images is performed to examine the amorphous and crystalline structures of the films at the atomic scale.

## Results and discussion

### Machine learning

We start the implementation of the model by selecting three sets of descriptors: 38 EDs with linear combinations of basic properties, 11 PDs, and CDs that encompass all EDs and PDs. For each set, we perform dimensionality reduction on the standardized descriptors using PCA. We retain *n* principal components and use this *n*-dimensional space as the input for the GPC. From GPC we obtain the prediction for the GFA probability *P*_glass_ as the posterior mean.

**Fig. 2:**
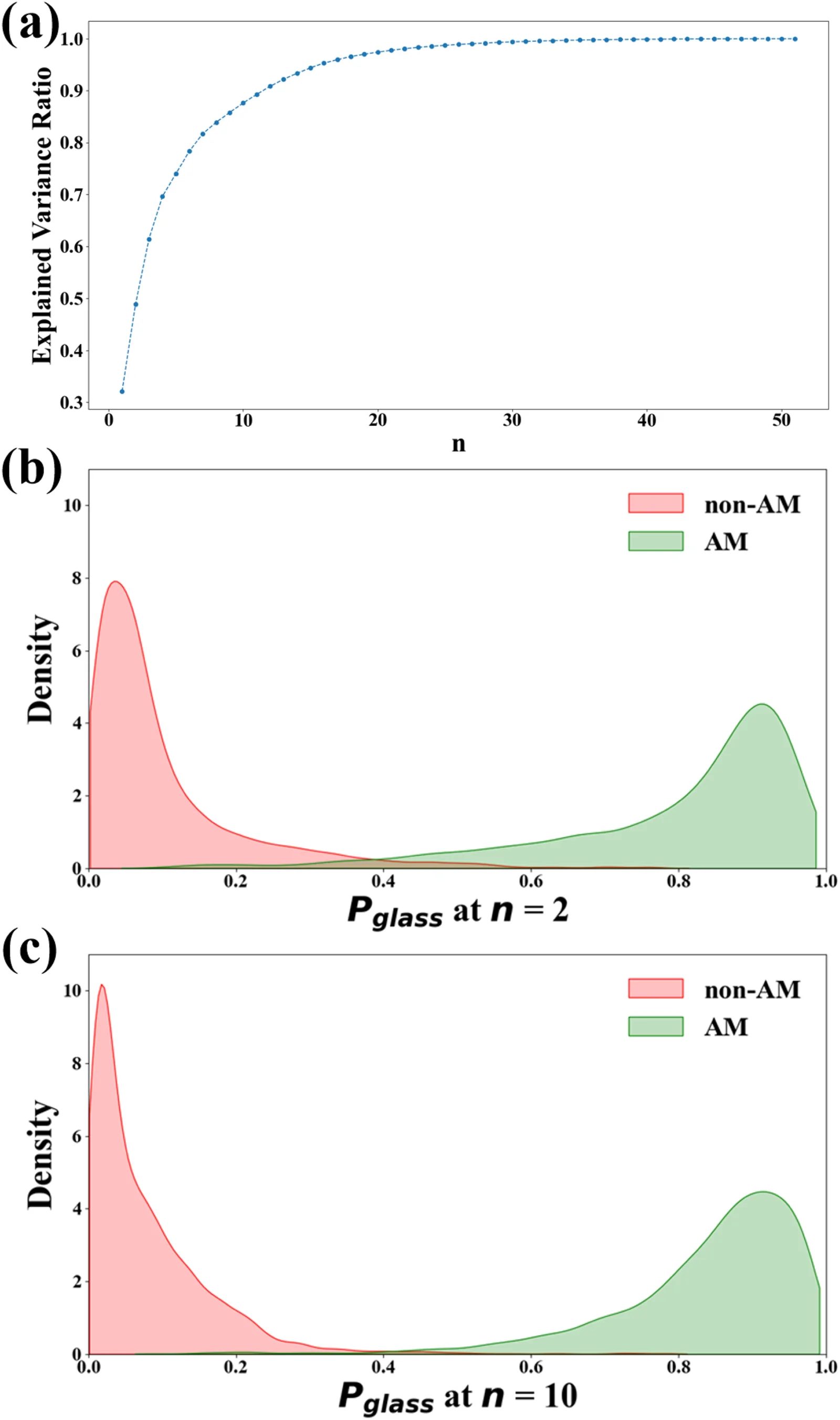
(a) The explained variance ratio of the PCA dimensionality reduction as a function of the reduced dimension *n* on CDs. (b) The probability distribution of the machine learning GFA predictions *P*_glass_ using EDs and *n* = 2 for all the compositions in the training set. The green plot shows the compositions corresponding to AM structures and red plot to the other structures. (c) Same as panel b but for *n* = 10.

Naturally, increasing *n* explains the higher proportion of the variance in the input data (Fig. 2a). We see that by using *n* = 7 we already explain 80% of the variance, demonstrating the effectiveness of the dimensionality reduction. By plotting the probability densities of *P*_glass_ for AM structures and combined non-AM structures, using *n* = 2 (Fig. 2b) and *n* = 10 (Fig. 2c) for the training set, we see that the model quite well captures the behavior already with low-dimensional input spaces. Both *n* values yield two separate peaks (one in the low *P*_glass_ region and one in the high *P*_glass_ region) with almost no overlap, indicating that the model effectively distinguishes between glass-forming and non-glass-forming compositions. Similar plots like those shown in Fig. 2 for different *n* values are presented in the SM (Fig. S1).

To further explore the effect of the dimensionality of the input space, we binarize the predictions. This is done by defining the prediction as true if the composition has AM structure and *P*_glass_ > 0.5, and *vice versa* for non-AM structures with *P*_glass_ < 0.5. Based on this binarization, we calculate four different scores for the predictions: the true positive rate using the true positives (TP) and false negatives (FN)6
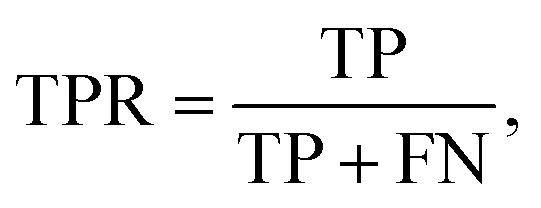
the true negative rate using true negatives (TN) and false positives (FP)7
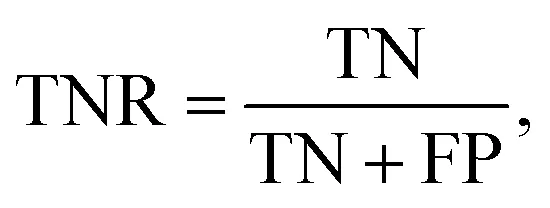
the false positive rate using false positives (FP) and true negatives (TN)8
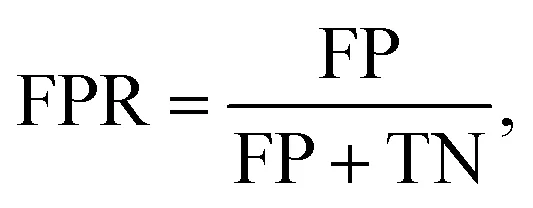
and the false negative rate9
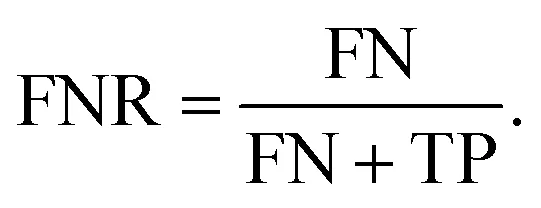
We must stress that our probabilistic prediction *P*_glass_ is more nuanced than this binarization which is merely an approximation for computing metrics that assess the prediction accuracy.

**Fig. 3:**
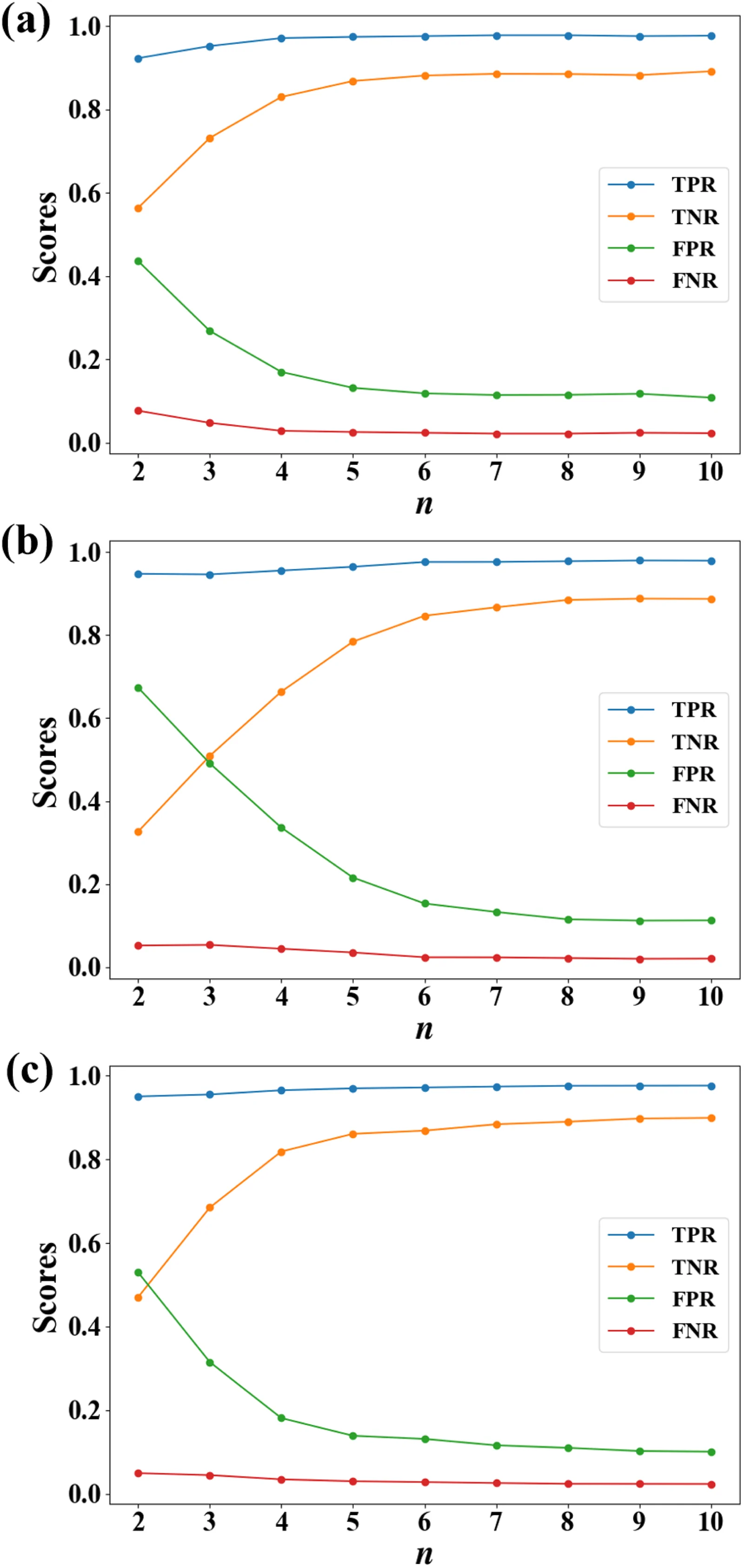
The prediction scores (eqn (6)–(9)) as a function of the number *n* of the principal components for (a) EDs, (b) PDs, and (c) CDs descriptor sets.

By computing these scores for different *n* values and for all three different descriptor sets (Fig. 3) we arrive at two key observations. Firstly, the scores expectedly improve when the dimensionality of the input space *n* is increased (consistently with the explained variance) but the scores saturate at around *n* = 7–9. Secondly, the EDs (Fig. 3a) perform better compared to PDs (Fig. 3b) and CDs (Fig. 3c), especially at low *n* values. The TPR and FNR scores are roughly equal for all descriptor sets, but in TNR and FPR scores we can see that the performance of PDs is clearly worse (especially for *n* < 7), CDs being only slightly better. Based on these observations, we use the EDs descriptor set for the analysis presented in the remainder of the article.

**Fig. 4:**
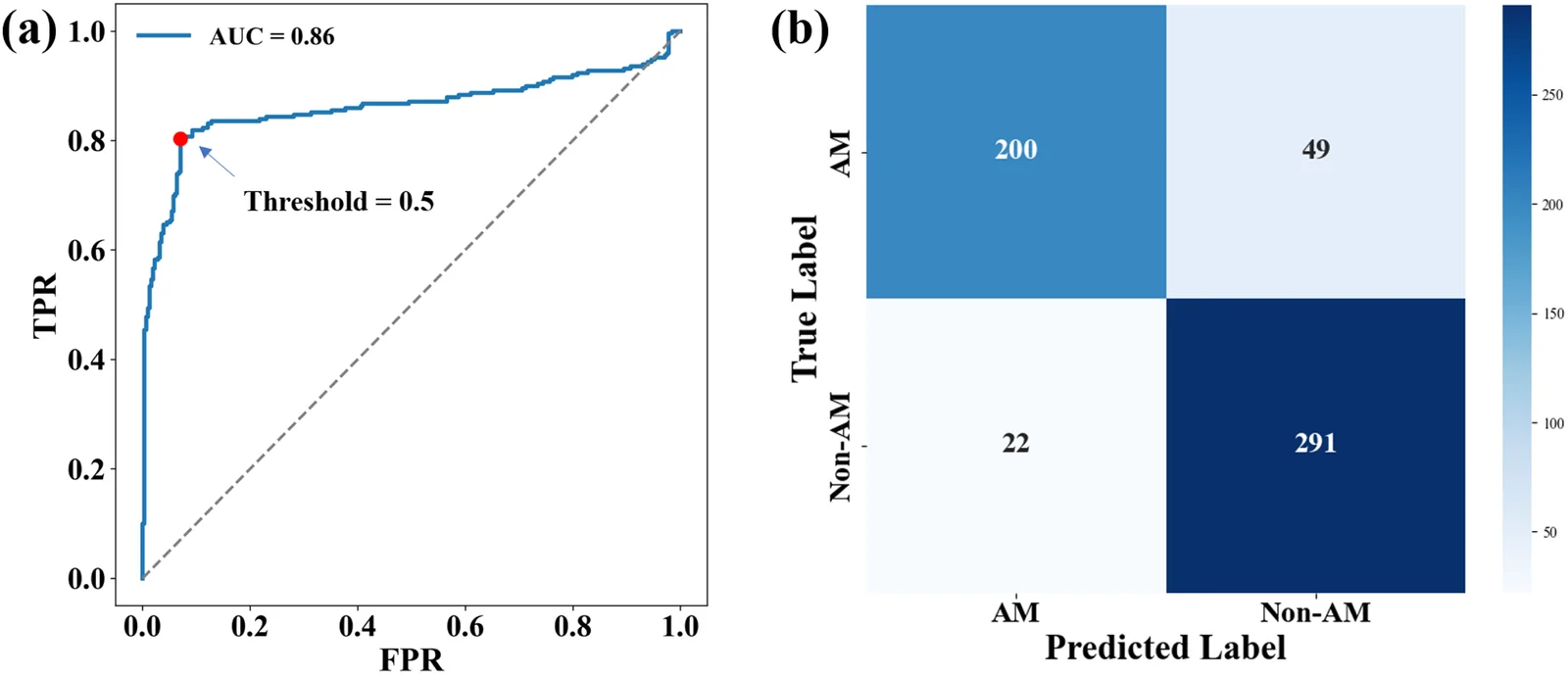
(a) The receiver operating characteristic (ROC) curve of the prediction results for the test set, using the EDs descriptor set with the model with the highest accuracy (*n* = 9). (b) The corresponding confusion matrix computed using the 0.5 threshold.

With the selected descriptor set (ED) and the optimized dimension of the input space (*n* = 9) we can test the prediction properties using an independent test set comprising 562 thin-film compositions. In Fig. 4a we show the receiver operating characteristic (ROC) curve. The EDs model achieves an area under the ROC curve (AUC) of 0.86, indicating the outstanding classification of the model. Equivalently, there is an 86% probability that the model assigns a higher *P*_glass_ to a randomly selected AM composition than to a randomly selected non-AM composition. As shown in Fig. 2b and c, the predicted probability distributions are well separated, making the probability threshold of approximately 0.5 a good decision boundary for classification. Fig. 4a highlights the operating point corresponding to a threshold of 0.5 using a red marker. At this threshold, the model correctly identifies most amorphous compositions while maintaining a low false positive rate. In Fig. 4b, using a decision threshold of 0.5, we show the confusion matrix of the binary prediction of the test set from which it follows that the model has an overall accuracy of 87% (defined as the percentage of correct predictions). The confusion matrix broken down by alloy order is presented in SM Fig. S3a, and the corresponding prediction accuracy as a function of alloy order is shown in Fig. S3b. Quaternary and quinary alloys account for only approximately 28% of the independent test set, some of these compositions remain compositionally close to ternary alloys, and from Fig. S4 it can be seen that the accuracy is quite dependent on the main element of the alloy. Therefore, the current results are insufficient to conclude that the model has strong performance for highly complex alloy systems. Nevertheless, the overall results demonstrate that the model achieves good predictive performance for previously unseen compositions.

To further evaluate the probabilistic output of the GPC model, the precision–recall (PR) analysis is performed by treating AM as the positive class and shown in SM Fig. S5. The model achieved an average precision (AP) of 0.89, higher than the random baseline 0.44 corresponding to the fraction of amorphous samples in the test set. The probability calibration of the GPC model is analyzed using a calibration curve (SM Fig. S6). The calibration curve shows that the predicted probabilities generally follow the observed positive frequencies, although moderate deviations from perfect calibration remain.

The lower performance on the test set is to be expected considering the machine learning nature of the GFA prediction model. Improving the prediction could be achieved by feeding experimentally observed data back to the model^38^ in an active learning framework, but this improvement would be mostly specific to the experimental system considered, rather than general.

**Fig. 5:**
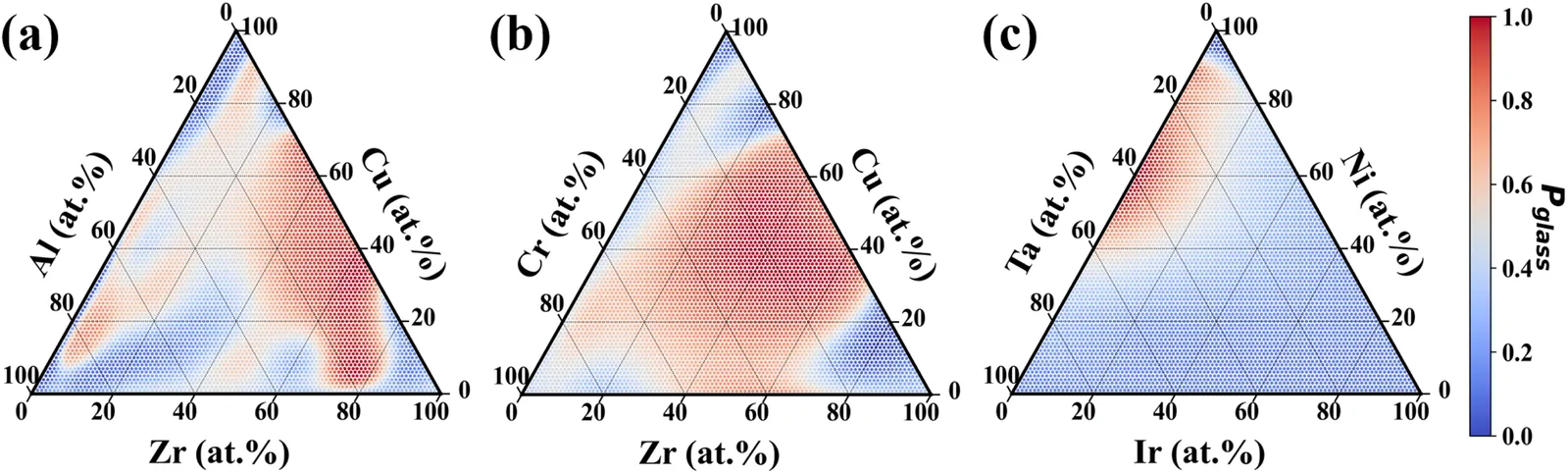
Maps of the GFA prediction *P*_glass_ in composition space for different ternary systems (a) Cu–Zr–Al, (b) Cu–Zr–Cr, and (c) Ni–Ta–Ir.

The trained and verified model can then be used to generate GFA maps in composition space for selected ternary compositions (Fig. 5). We have chosen three archetypal TFMG systems, Cu–Zr–Al (Fig. 5a), Cu–Zr–Cr (Fig. 5b), and Ni–Ta–Ir (Fig. 5c), and generated GFA predictions with a 1% composition interval, to compare with the results reported in the literature.^36^ Among the three systems, only the Cu–Zr–Al system exists in the training set, with a total of 13 samples. Cu–Zr–Al shows a region of high *P*_glass_ around equiatomic Cu–Zr with 0–20% Al addition.^62^ Our prediction however gives high GFA also for very Zr-rich compositions. Interestingly, our model also predicts moderately high GFA for small windows of Al-rich compositions, especially with low Zr content. For Cu–Zr–Cr the highest predicted GFA is similarly achieved near equiatomic Cu–Zr, but the Cr addition can range up to around 40%.^63^ For Ni–Ta–Ir the predicted window for high GFA is around Ni_0.6_Ta_0.4_ with very small amounts of added Ir (less than 10%).^64^ Overall, the predicted high-GFA regions are broadly consistent with the composition ranges expected from previous studies,^36^ which confirms the accuracy of our model.

### Descriptor importance

**Fig. 6:**
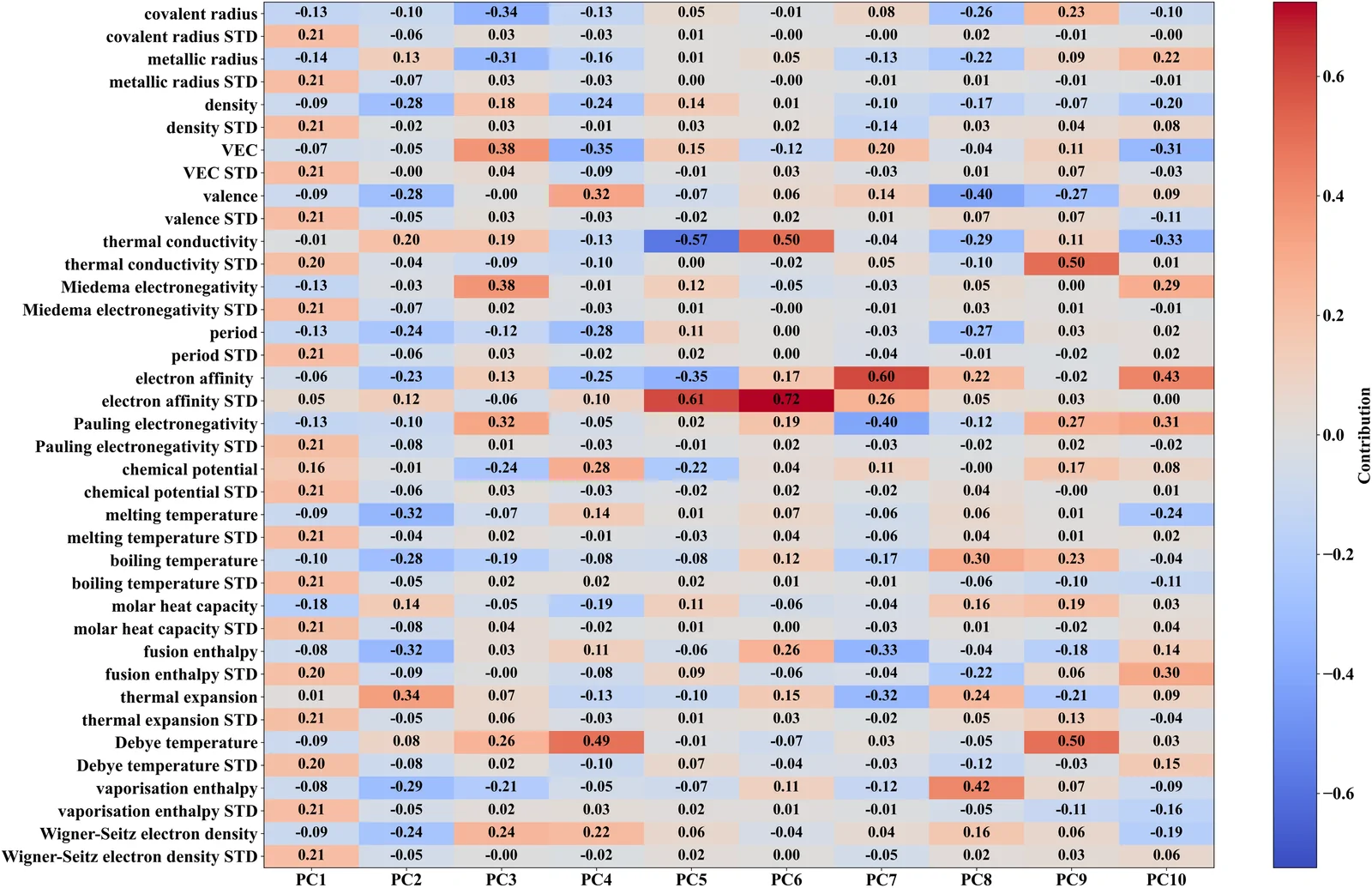
The weights (shown by the color) of the (standardized) input descriptors (rows) in the different principal components (columns) when EDs are used. The STD refers to the standard deviation of the descriptor (eqn (2)).

For the EDs with the highest prediction accuracy, we examine the principal component (PC) weights and visualize the results in the heatmap shown in Fig. 6. The numbers in the heatmap represent the coefficients in the linear combination defining the PCs (columns) based on the original standardized descriptors (rows). Dimension PC1, which explains 36% of the variance in the data (Fig. 2a), is equally weighted by all of the standard deviation (STD) descriptors (except electron affinity), favoring thus compositional disorder in the properties. Going through the other principal components and their dominant contributions: PC2 captures the thermodynamic property opposition between thermal expansion (positive contribution) and melting temperature (negative contribution). This reveals a material design relationship, that is, high heat resistant materials often have low thermal expansion coefficients.^65^ PC3 has positive contributions from electronic structure parameters (VEC, electronegativities) and negative contributions from the atomic radii, reflecting bonding compactness. PC4 has strong positive contribution from the Debye temperature, as well a smaller one from valence, and a negative contribution from VEC. This means a positive contribution from lattice stiffness and strong bonding, and a negative contribution from electron-rich systems. Above 4 PCs, the increase in the accuracy of the predictions is much slower (Fig. 3). Both PC5 and PC6 have strong contributions from the standard deviation of electron affinity and thermal conductivity. In PC5 the contributions have opposing signs, but in PC6 they both have a positive contribution. In PC7 a positive contribution comes from electron affinity and a negative contribution from Pauling electronegativity. This would separate elements that easily attract electrons but have low bond polarity from ones exhibiting more polar or ionic bonding.

In general, retaining the nine first PCs (*n* = 9) results in the highest prediction precision. While individual EDs descriptors exhibit varying weights to each component, their cumulative contributions across the first nine PCs (properly weighted by the explained variance ratio of each principal component, see Fig. 2a) are relatively evenly distributed (weights 0.13 ± 0.01). This means that all descriptors play a role in determining the GFA, even though some of them might be strongly correlated and thus redundant. To compare the contributions of EDs and PDs to the PCA representation, we also analyzed the PCA loadings of the CDs descriptor set, and the results are presented in SM Fig. S7.

Our approach also enables another way to look at the importance of the descriptors, and their combinations in the PC-space. We can do this by looking at the corresponding length scales of the RBF kernel. Due to the GPC methodology used, there are separate length scales for each class (see SM, SI Table III, for a list of all length scales). We can see that, *e.g.*, for prediction of AM structures, the shortest length scales are associated with PC-7 and PC-2, so differences in these principal component directions yield the strongest differences in the prediction. The same analysis shows that PC-7 is nearly irrelevant for predicting CR structures, as the enormous length scale makes the GP in this direction practically constant.

It should be noted that these length scales do not directly reflect the feature importance ranking of the descriptors. Each principal component is a linear combination of many descriptors, and therefore the length-scale analysis mainly reflects model sensitivity in the retained PCA space. To further analyze the importance of the specific descriptors, one would normally use methods such as permutation importance. It refers to randomly permuting the training data for a specific descriptor, effectively rendering it meaningless for the prediction, and seeing how much the prediction score decreases. However, as our workflow includes a dimensionality reduction step, this method cannot be effectively used here. As the permutation of a single descriptor also affects the PCA, it indirectly has an influence on the other descriptors due to the change in the PCs. Thus, assessing the effect of changing a single descriptor becomes extremely challenging.

We observe that the EDs perform better than PDs or CDs. However, PDs have previously been successfully used as a GFA indication, especially in BMGs.^46,48^ The use of PDs alone describes a simplified physical model of the key features of GFA, but is seemingly not sufficient when TFMGs are considered. We find EDs, simplified descriptors of the composition, more useful when a large composition space is considered. When PDs are incorporated, their contribution to PCA is substantial. However, this contribution negatively impacts model performance, leading to a decrease in accuracy when PDs are combined with EDs (*i.e.*, CDs). In far-from-equilibrium synthesis scenarios—including the high cooling rates achieved in magnetron sputtering—the use of equilibrium thermodynamic descriptors, such as mixing enthalpy,^66^ is not as suitable compared to more simplified descriptors.

Our test set also includes quaternary and quinary alloy compositions, accounting for about 28% of the total test set. Our over 6000 training samples are all binary and ternary compositions. Due to the more complex composition relationships (cocktail effects) in quaternary and higher order systems, the feature distribution of some samples may exceed the feature space covered by the training data, thereby forcing the model to extrapolate beyond the parts of the descriptor space it was trained for. Using Gaussian processes with stationary kernels, the model can only extrapolate around one kernel lengthscale 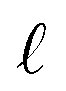 away from the points in the training set. However, we have checked that the lengthscales far exceed the distance between training and test set points in the PCA-reduced descriptor space.

Based on the analysis of the PC weights, the key descriptors of our model are related to compositional heterogeneity, elementary thermodynamic properties, electronic structure parameters, and atomic radii. This qualitatively aligns with Inoue's classical empirical rules.^62^ In contrast, descriptors such as Δ*S*_ideal_, Δ*H*_mix_ and the bulk modulus play a more decisive role in the design of BMGs. This proves that our model has indeed learned the latent physical rules.

### Experimental verification

**Fig. 7:**
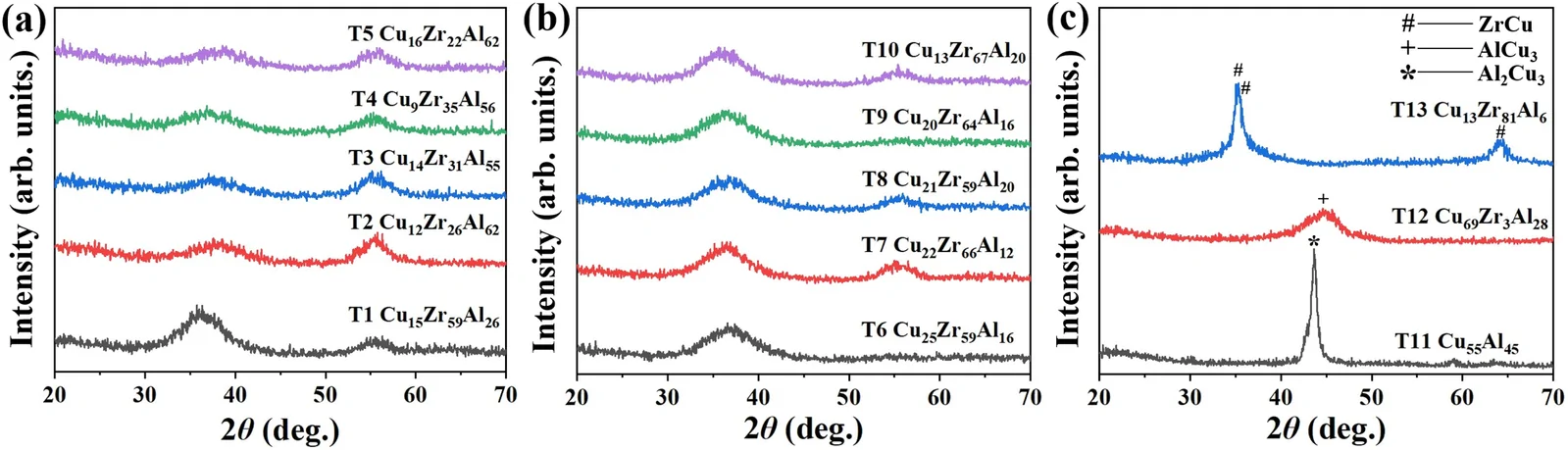
Initial GIXRD patterns of all the samples: (a) T1–T5 are low *P*_glass_ (<0.5) samples, (b) T6–10 are high *P*_glass_ (>0.5) samples, and (c) T11–13 are low *P*_glass_ samples with compositions close to those of binary alloys. The corresponding peaks of binary alloys are illustrated in the figure and the alloys shown in the legend.

To experimentally verify our BC-based *P*_glass_ prediction (Fig. 5a), we synthesize different thin-film compositions within the ternary Cu–Zr–Al system using magnetron sputtering, and study the sample microstructure and phase composition using GIXRD. A total of 13 thin-film samples are prepared as detailed in Table 3. The compositions are chosen so that 8 of the samples have a predicted *P*_glass_ below 0.5, while 5 of the samples have *P*_glass_ values above 0.5, ensuring a representative distribution across different *P*_glass_ values. Table 3 also lists the predicted *P*_glass_ values from the BC model for the synthesized samples. The GIXRD patterns for the as-deposited samples are shown in Fig. 7. Most samples exhibit a wide and diffuse scattering peak between 2*θ* = 30° and 50° (Fig. 7a and b, samples T1–T10) indicating the formation of an amorphous structure at the high cooling rates prevailing during the thin-film deposition process. Only a few samples are exceptions to that trend, with compositions very close to those of binary alloys exhibiting crystalline peaks (Fig. 7c). As shown in Fig. 7c, the identified phases are Al_2_Cu_3_ (PDF 25-0015)^67^ for T11 (Cu_55_Zr_0_Al_45_), AlCu_3_ (PDF 28-0005)^68^ for T12 (Cu_69_Zr_3_Al_28_), and ZrCu (PDF 22-0233)^69^ for T13 (Cu_13_Zr_81_Al_6_).

**Table 3:** BC predicted *P*_glass_ of magnetron sputtered Cu–Zr–Al samples and the final structure after annealing

Sample	Composition (at%)	*P*_glass_ (%)	As-deposited structure	Final structure
T1	Cu_15_Zr_59_Al_26_	42	AM	AM
T2	Cu_12_Zr_26_Al_62_	12.7	AM	CR
T3	Cu_14_Zr_31_Al_55_	16	AM	CR
T4	Cu_9_Zr_35_Al_56_	24	AM	CR
T5	Cu_16_Zr_22_Al_62_	20	AM	CR
T6	Cu_25_Zr_59_Al_16_	84	AM	AM
T7	Cu_22_Zr_66_Al_12_	87	AM	AM
T8	Cu_21_Zr_59_Al_20_	75	AM	AM
T9	Cu_20_Zr_64_Al_16_	84	AM	CR
T10	Cu_13_Zr_67_Al_20_	78	AM	AM
T11	Cu_55_Zr_0_Al_45_	20	CR	CR
T12	Cu_69_Zr_3_Al_28_	26	CR	CR
T13	Cu_13_Zr_81_Al_6_	42	CR	CR

The data presented in Fig. 7 match previous experimental results,^36,70–73^ but they differ from our GFA predictions (*P*_glass_). This underpins the fact that the ML model predictions depend crucially on the experimental synthesis methods and parameters, as also observed in previous studies.^38^ Magnetron sputtering exhibits a multitude of process parameters, including working pressure, target-to-substrate distance, and deposition temperature, the selection of which may need to be optimized for matching experimental data with model predictions.

To understand the effect of cooling rate on the structure formation of the sputtered Cu–Zr–Al films, we anneal the as-deposited samples as a way to resemble conditions of lower cooling rate compared to that prevailing during vapor condensation. Annealing experiments are conducted according to the description provided in the Methods section.

**Fig. 8:**
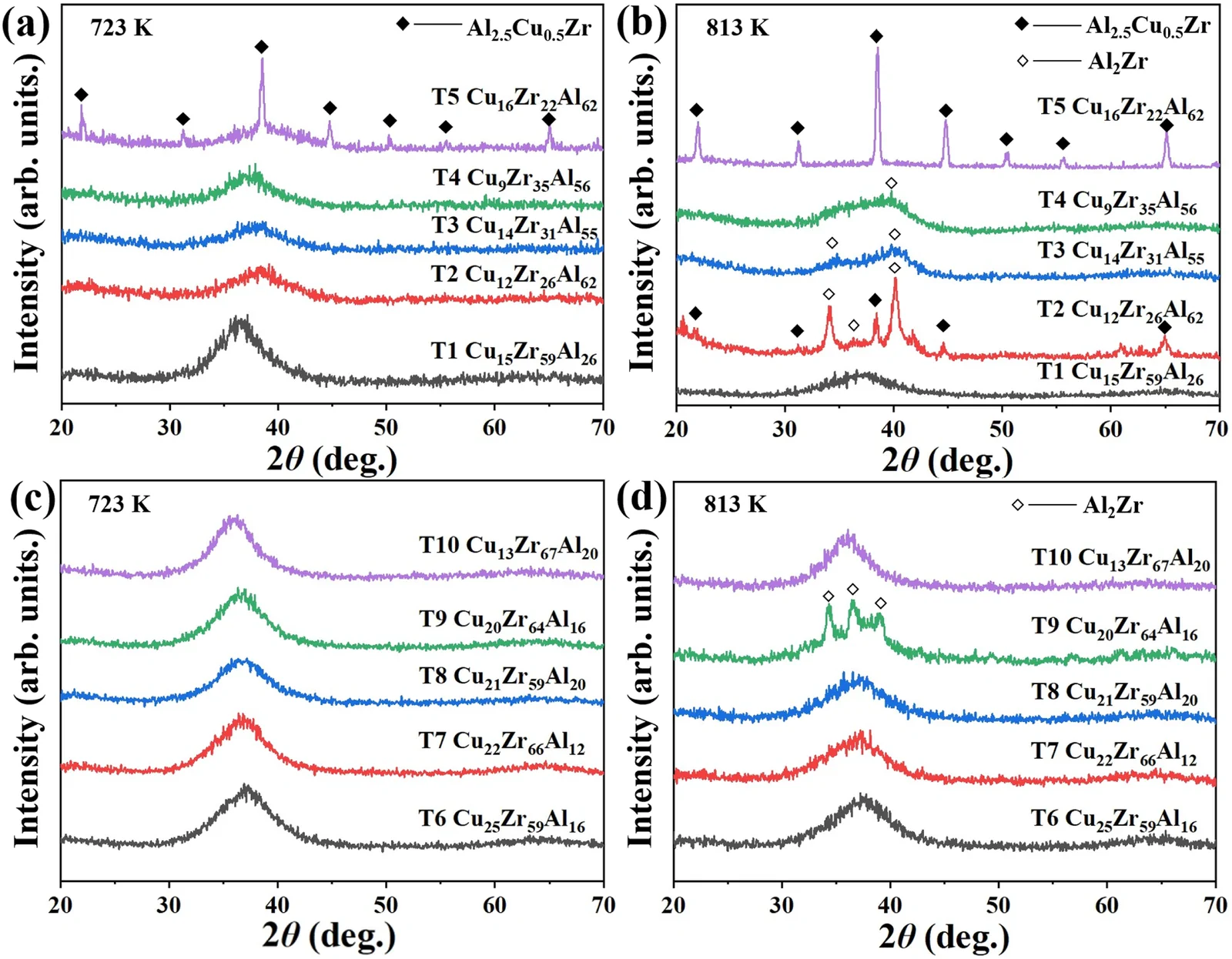
GIXRD patterns after annealing the low *P*_glass_ samples T1–T5 up to (a) 723 K and (b) 813 K, using 1 h annealing with 30 K temperature increments. Same results also for the high *P*_glass_ samples T6–T10 for (c) 723 K and (d) 813 K, using the same process. The peaks in the figures have been identified with the corresponding alloys shown in the legend.

Since samples T11–T13 are already crystalline in the as-deposited state, they are not subjected to annealing experiments. The GIXRD patterns after annealing low *P*_glass_ samples T1–T5 at 723 and 813 K are shown in Fig. 8a and b, respectively. After 1 h at 723 K, a sharp peak appears on the diffuse scattering peak of T5, while the other samples do not exhibit any changes. When the temperature is increased up to 813 K, the diffuse scattering peaks of T2 and T5 completely disappear, and the diffraction pattern is dominated by sharp peaks that indicate the formation of a crystalline structure. Signs of crystallization are also seen in T3 and T4, while T1 still maintains its originally amorphous structure. Most of the samples with higher *P*_glass_ values (T6–T10) maintain an amorphous structure after annealing at 813 K, and only sample T9 has a crystalline peak. The diffraction peaks seen in the patterns of the annealed samples can be attributed to Al_2_Zr (PDF 13-0510)^74^ for T2, T3, T4, and T9, and to Al_2.5_Cu_0.5_Zr (PDF 47-1297)^75^ for T5. Additional GIXRD patterns of the samples annealed up to 723 K, 753 K, 783 K, and 813 K are shown in SM Fig. S8.

To further analyze the microstructure of the thin-films, based on the XRD results, the samples T4 (Cu_9_Zr_35_Al_56_) and T8 (Cu_21_Zr_59_Al_20_) annealed at 813 K are selected for TEM analysis. Sample T8 remains amorphous after annealing, while sample T4 exhibits the weakest tendency towards crystallization among the samples that undergo microstructural changes upon heat treatment.

**Fig. 9:**
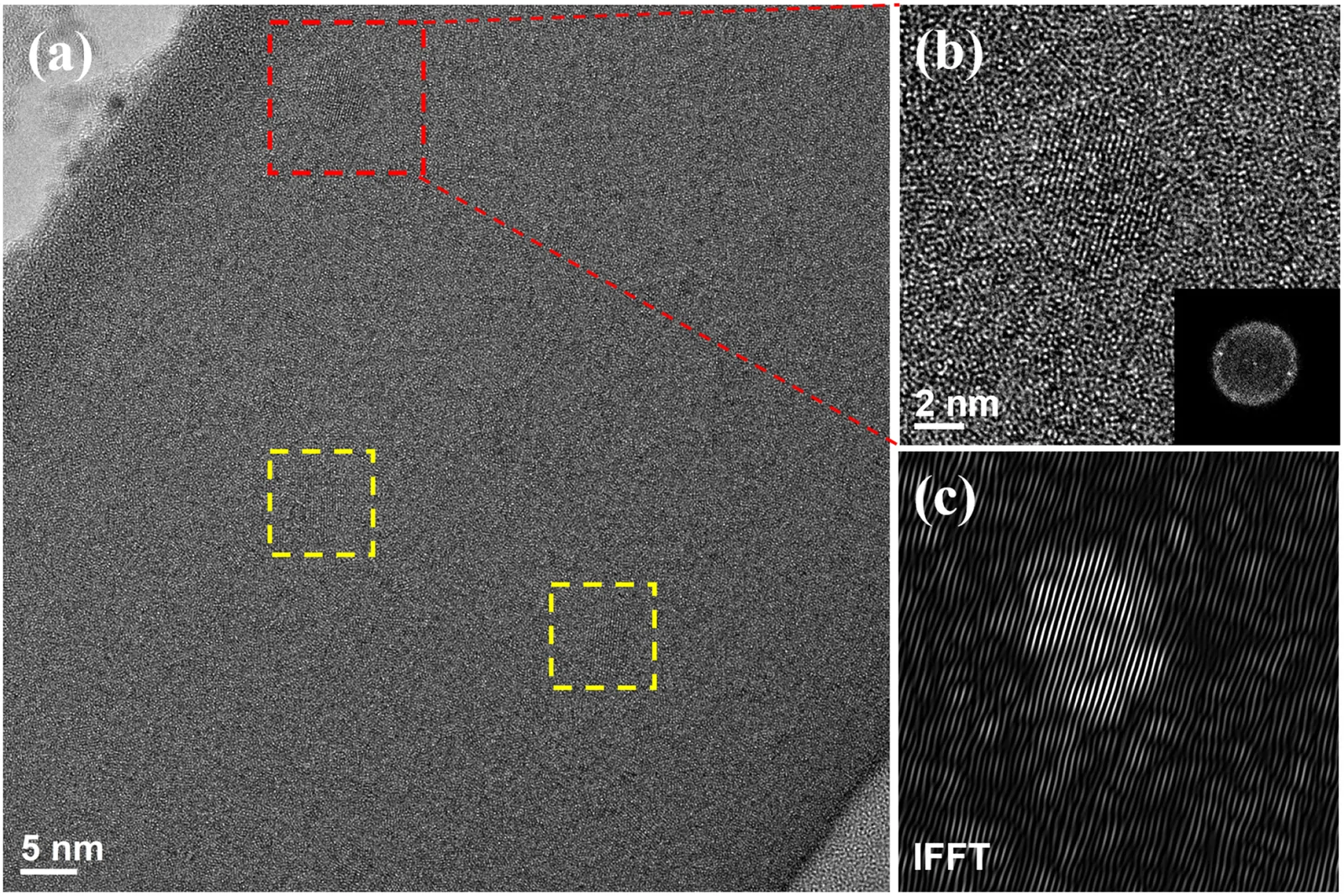
Cross section HRTEM image of (a) T4 (Cu_9_Zr_35_Al_56_), ordered structures can be observed in the sample, the nanocrystals highlighted by the dashed boxes all exhibit a *d*-spacing 2.6 Å; (b) magnified view of the red dashed area with FFT inset and (c) its IFFT, showing the lattice fringes signal.

Fig. 9a shows an HRTEM image of the T4 film, which comprises nanosized ordered crystalline regions embedded in an amorphous matrix. Three representative areas are highlighted by the dashed squares. Fig. 9b presents a magnified view of the red dashed squares, and the FFT pattern is shown in the bottom right inset, where distinct crystalline spots are visible together with the diffuse rings. Fig. 9c shows the inverse fast Fourier transform (IFFT) of Fig. 9b, in which clear lattice fringes are observed, the *d*-spacing of the fringes is approximately 2.6 Å, which is close to the (110) planes in Al_2_Zr phase.^74^ This observation is consistent with the weak crystallization signal observed in the XRD pattern of T4, confirming that the matrix consists of a mixture of nanocrystalline and amorphous phases.

**Fig. 10:**
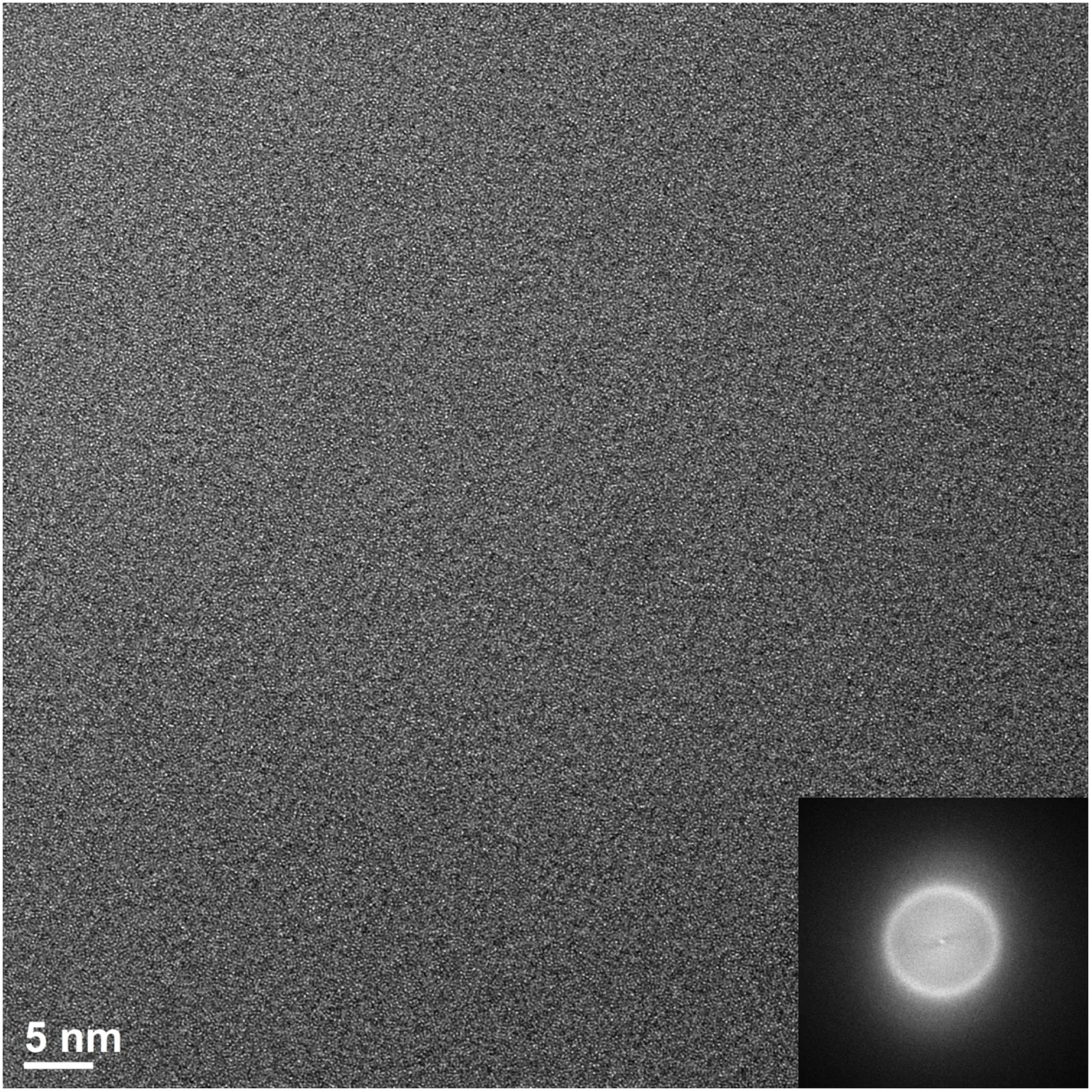
Cross section HRTEM image of T8 (Cu_21_Zr_59_Al_20_), which is amorphous, global FFT is shown in the inset.

Fig. 10 presents the HRTEM image of the T8 sample. No clear crystalline structure is observed. The FFT pattern shows diffuse rings without any discernible spots, which is consistent with the broad amorphous peak observed in the XRD pattern. These results confirm that the T8 film remains fully amorphous after annealing.

More TEM images taken from multiple regions of the samples—including the film surface, the film bulk, and the film–substrate interface—are displayed in SM Fig. S9 and S10. The results are consistent with the XRD analysis: T8 exhibits a homogeneous amorphous structure, whereas T4 shows a mixed amorphous–nanocrystalline structure, further confirming the agreement between the model predictions and the experimental validation results.

**Fig. 11:**
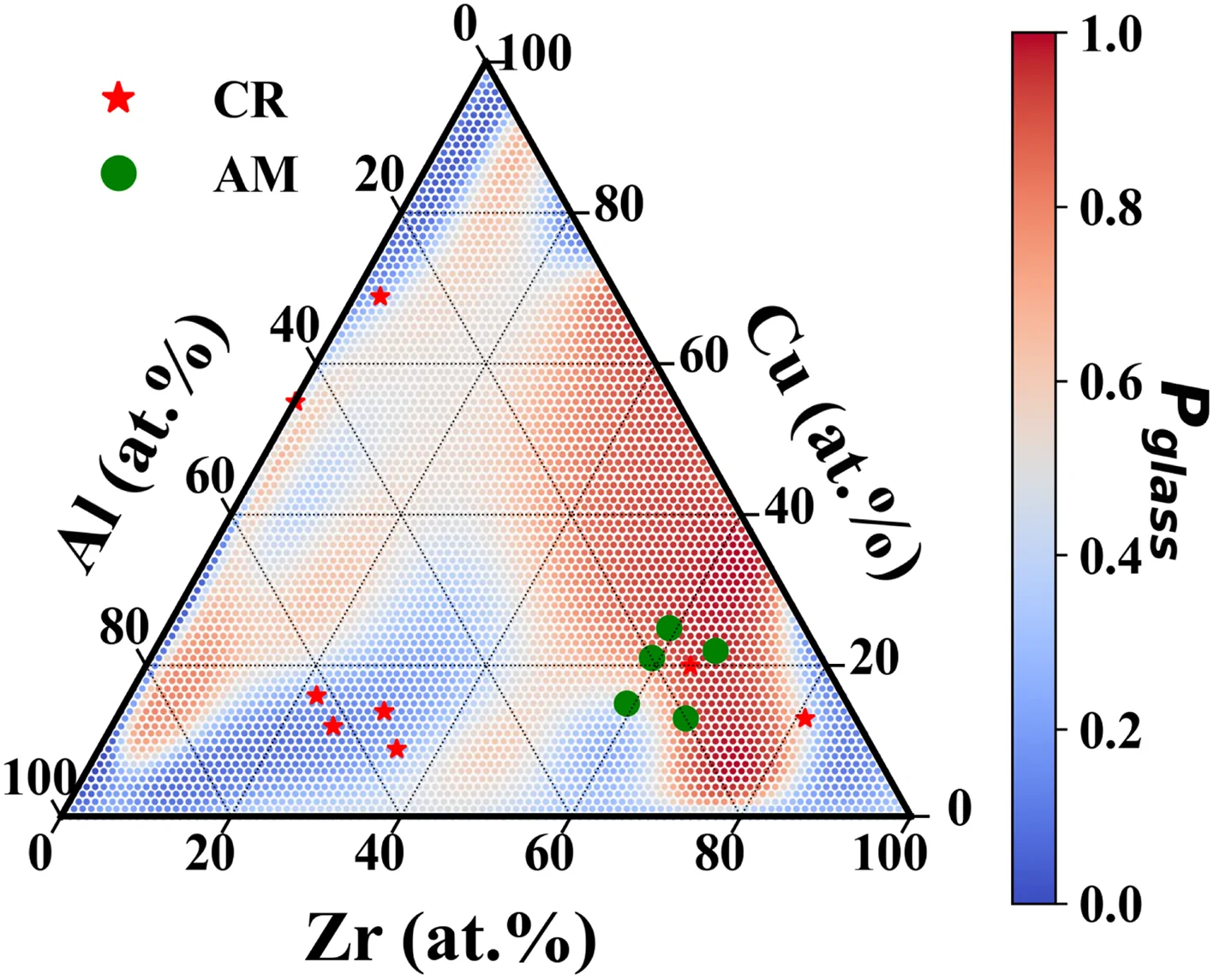
Experimentally determined structure of annealed samples (CR or AM) superimposed on the predicted GFA map.

The structures of all samples, including those after annealing and those originally crystalline (T11–T13), *i.e.*, CR or AM (marked with red stars and green circles, respectively), are illustrated in Fig. 11, superimposed on the predicted *P*_glass_ values. As can be seen, amorphous structures are retained in the moderate Al concentrations (<30%) and with compositions not dominated by Cu or Zr. Only sample T9 (Cu_20_Zr_64_Al_16_) is an outlier. It has a high predicted *P*_glass_ and resides in a cluster of amorphous samples, but showed clear crystallization upon annealing. We can thus say that the experimental results are roughly consistent with the model, high *P*_glass_ indicating good GFA. We also know that the compositions in the middle of our largest cluster of high *P*_glass_ (along the 10% Al line) have high GFA, based on the results of ref. 36.

## Summary and conclusions

In the present work, we use literature data, and descriptors computed based on the compositions, to train a GPC-based machine learning model to predict the GFA of TFMGs. The prediction results are then experimentally verified by synthesizing samples using magnetron sputtering, and characterizing them.

We use three different descriptor sets (ED, PD, and CD) for training the model. In the ultra-high cooling rates used in magnetron sputtering, the PDs based on equilibrium thermodynamic quantities—and CDs including them—perform worse than the simplified EDs. The best accuracy achieved with EDs on an independent test set was 87%.

Our experimental results show that magnetron sputtering yields amorphous structures in a much wider composition range than our model predicts. Through thermal annealing of as-deposited samples, we mimic indirectly synthesis conditions with lower cooling rates, and show that our prediction results align well with the true GFA. Our ML predictions can then provide a good reference for compositional search and alloy design.

The GPC-based machine learning framework is thus a good platform for accelerating the exploration of GFA. In the future, guided by the model developed in this study, we will use pulsed sputtering experiments to investigate the medium-range order in metallic glasses. In addition, Bayesian and Gaussian process model performs well on small datasets and are expected to be further improved for the prediction of GFA in bulk metallic glasses.

## Author contributions

Xuliang Luo: writing – original draft, data collection, machine learning investigation, experiments, and characterization. Tero Mäkinen: writing – review & editing, data collection, software, machine learning investigation, methodology, conceptualization. Wenyi Huo: writing – review & editing, investigation (experiments), formal analysis, methodology, conceptualization. Silvia Bonfanti: writing – review & editing, investigation (machine learning). Zhehao Chen: investigation (experiments), writing – review & editing. Kenichiro Mizohata: investigation (experiments), writing – review & editing. Kostas Sarakinos: writing – review & editing, supervision, investigation (experiments), methodology. Mikko J. Alava: writing – review & editing, supervision, methodology, conceptualization.

## Conflicts of interest

The authors declare that they have no known competing financial interests or personal relationships that could have appeared to influence the work reported in this paper.

## Supplementary Material

RA-OLF-D6RA07674B-s001

## Data Availability

The dataset and original code that support the findings of this study are available in Zenodo at https://doi.org/10.5281/zenodo.21969413. Supplementary information (SI) is available. See DOI: https://doi.org/10.1039/d6ra07674b.
