# Supplementary material for: Data-driven prediction of thin-film metallic glass forming ability via Bayesian classification and experimental verification in the Cu–Zr–Al system
Source: RSC Adv. 2026 Sep 11;16(43):54061–75. doi: 10.1039/d6ra07674b (PMC13563395; doi:10.1039/d6ra07674b)
Supplement: Supplementary file 1 [file RA-016-D6RA07674B-s001.pdf]

Supplementary Information (SI) for RSC Advances.
This journal is © The Royal Society of Chemistry 2026

Supplementary Material: Data-Driven Prediction of Thin-Film Metallic Glass Forming Ability via Bayesian Classification and Experimental Verification in the Cu-Zr-Al System

Xuliang Luo[1], Tero Mäkinen[1], Wenyi Huo[2], Silvia Bonfanti[2,3],
Zhehao Chen[4], Kenichiro Mizohata[4], Kostas Sarakinos[4,5], Mikko Alava[1]
[1] *Department of Applied Physics, Aalto University, PO Box 11000, 00076 Aalto, Espoo, Finland*
[2] *NOMATEN Centre of Excellence, National Center for Nuclear Research, 05-400, Otwock, Poland*
[3] *Center for Complexity and Biosystems, Department of Physics 'Aldo Pontremoli', University of Milan, Milano, Italy*
[4] *Department of Physics, University of Helsinki, PO Box 43, FI-00014, Helsinki, Finland*
[5] *KTH Royal Institute of Technology, Department of Physics, Roslagstullsbacken 21, 114 21 Stockholm, Sweden*
(Dated: August 16, 2026)

Ideal entropy	$\Delta S_{\text{ideal}} = -\sum c_i \ln c_i$
Xia's ideal entropy	$\Delta S_{\text{Xia}} = -\sum c_i \ln\left(\frac{x_i^3}{\sum_j c_j x_j^3}\right)$
Mismatch entropy	$\Delta S_{\text{mismatch}} = \frac{3}{2}(\zeta^2-1)y_1 + \frac{3}{2}(\zeta^2-1)y_2 - \left[\frac{1}{2}(\zeta-1)(\zeta-3)+\ln\zeta\right](1-y_3)$
	where $\zeta = \frac{25}{9}$
	and $y_1 = \frac{1}{\sum c_i x_i^3} \sum_{j>i}(x_i+x_j)(x_i-x_j)^2 c_i c_j$
	$y_2 = \frac{\sum c_i x_i^2}{(\sum c_i x_i^3)^2} \sum_{j>i} x_i x_j (x_i-x_j)^2 c_i c_j$
	$y_3 = \frac{(\sum c_i x_i^2)^3}{(\sum c_i x_i^3)^2}$
Mixing entropy	$\Delta S_{\text{mix}} = \Delta S_{\text{ideal}} + \Delta S_{\text{mismatch}}$
Mixing enthalpy	$\Delta H_{\text{mix}} = \sum_{i\neq j} c_i c_j H_{ij}$
Mixing Gibbs free energy	$\Delta G_{\text{mix}} = \Delta H_{\text{mix}} - T_m \cdot \Delta S_{\text{mix}}$
Lattice distortion	$d = \sum_{j\geq i} \frac{c_i c_j \lvert x_i + x_j - 2\bar{x}\rvert}{2\bar{x}}$
Mixing P_{HS}	$P_{\text{HS}} = \Delta H_{\text{mix}} \cdot \Delta S_{\text{mismatch}}$
Mixing P_{HSS}	$P_{\text{HHS}} = \Delta H_{\text{mix}} \cdot \Delta S_{\text{mismatch}} \cdot \Delta S_{\text{ideal}}$
Bulk modulus	$k = \sum c_i k_i$
Standard deviation of bulk modulus	$k_d = \sqrt{\sum c_i (k_i - k)^2}$

SUPPLEMENTARY TABLE I. Formulas for calculating physical descriptors, where x_i is metallic radius of ith element, H_{ij} is the mixing enthalpy between element i and j, T_m refers to melting temperature, k_i is the bulk modulus of ith element.

**Target**	**Power / Current (W) / (mA)**	**Thickness (Å)**	**Deposition Rate (atoms cm^{-2} s^{-1})**	**Fitting Function**
Cu	54 W	223.86	$4.75 \cdot 10^{14}$	$R = 9.54 \cdot 10^{12} \cdot P - 4.3 \cdot 10^{12}$
	60 W	283.47	$6.02 \cdot 10^{14}$	
	80 W	365.17	$7.75 \cdot 10^{14}$	
	94 W	417.48	$8.86 \cdot 10^{14}$	
	104 W	462.18	$9.81 \cdot 10^{14}$	
Al	101 W	223.57	$3.37 \cdot 10^{14}$	$R = 3.47 \cdot 10^{12} \cdot P - 2.18 \cdot 10^{13}$
	111 W	234.52	$3.53 \cdot 10^{14}$	
	120 W	258.83	$3.90 \cdot 10^{14}$	
	140 W	312.12	$4.70 \cdot 10^{14}$	
	160 W	352.66	$5.31 \cdot 10^{14}$	
Zr	154 mA	153.74	$1.65 \cdot 10^{14}$	$R = 1.12 \times 10^{12} \cdot I - 8.52 \times 10^{12}$
	185 mA	183.76	$1.98 \cdot 10^{14}$	
	217 mA	215.34	$2.32 \cdot 10^{14}$	
	249 mA	253.14	$2.72 \cdot 10^{14}$	

SUPPLEMENTARY TABLE II. Sputtering deposition parameters for Cu, Al, and Zr. The thickness were measured by XRR, and the atomic deposition rates were estimated based on the density. The table also includes the linear fits between the deposition rate R and the target power P. For the DC power supply of Zr, the deposition rate was calculated as a function of the current I.

**Direction**	ℓ_{CR}	ℓ_{AC}	ℓ_{AM}
PC 1	1.54	6.18	1.45
PC 2	1.21	7.90	1.07
PC 3	1.40	1.22	1.52
PC 4	1.83	1.12	1.80
PC 5	1.08	3.30	1.47
PC 6	1.90	2.70	1.33
PC 7	766.20	1.77	0.86
PC 8	0.89	9.93	2.22
PC 9	1.48	1.45	1.22

SUPPLEMENTARY TABLE III. The RBF kernel lengthscales ℓ for CR, AC, and AM (columns) for each direction in PC-space (rows).

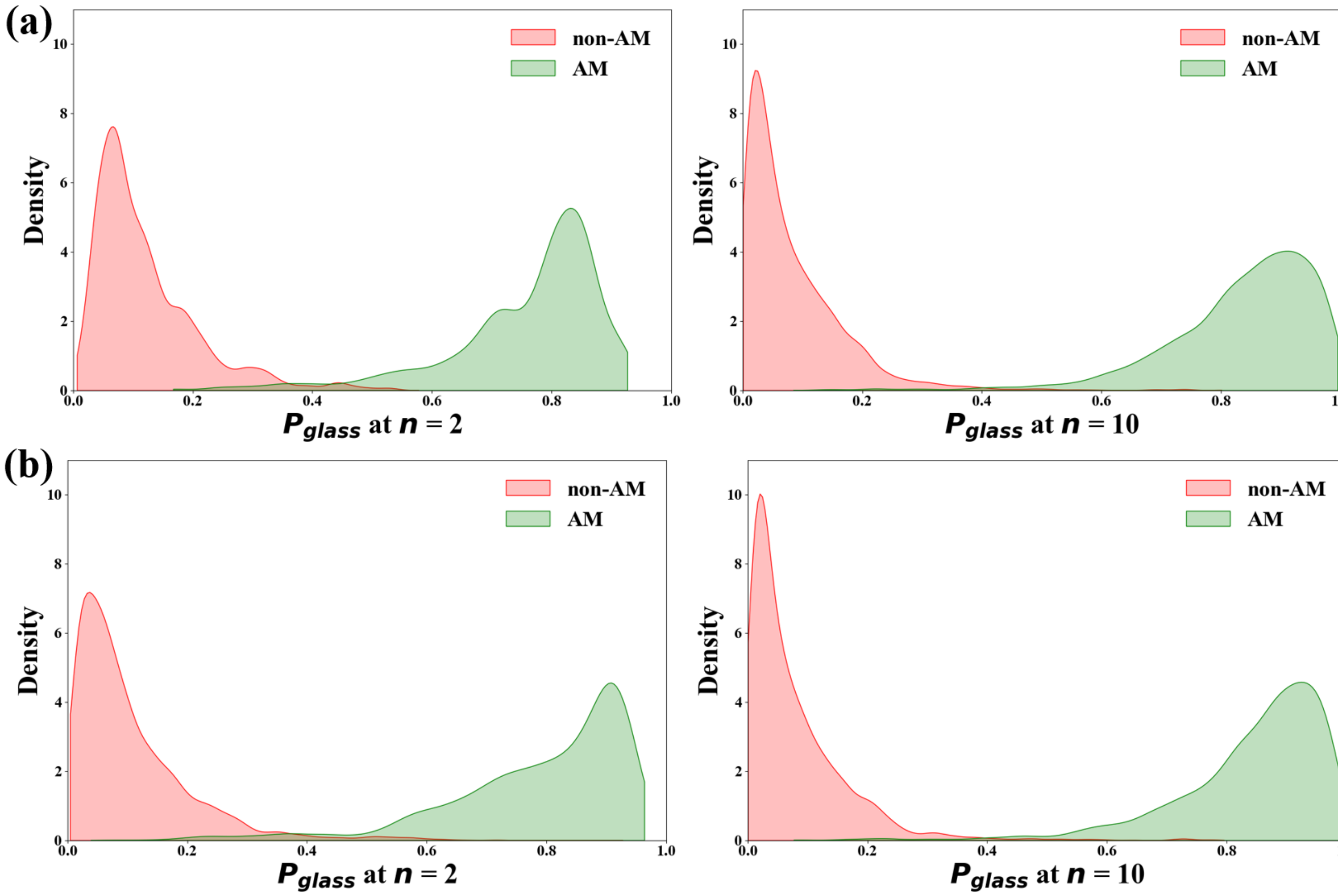

FIG. S1. The probability distribution of the machine learning GFA predictions P_{glass} for $n=2$ and $n=10$ using a) PD and b) CD descriptor set for all the compositions in the training set. The green plot shows the compositions corresponding to AM structures and red plot to the other structures.

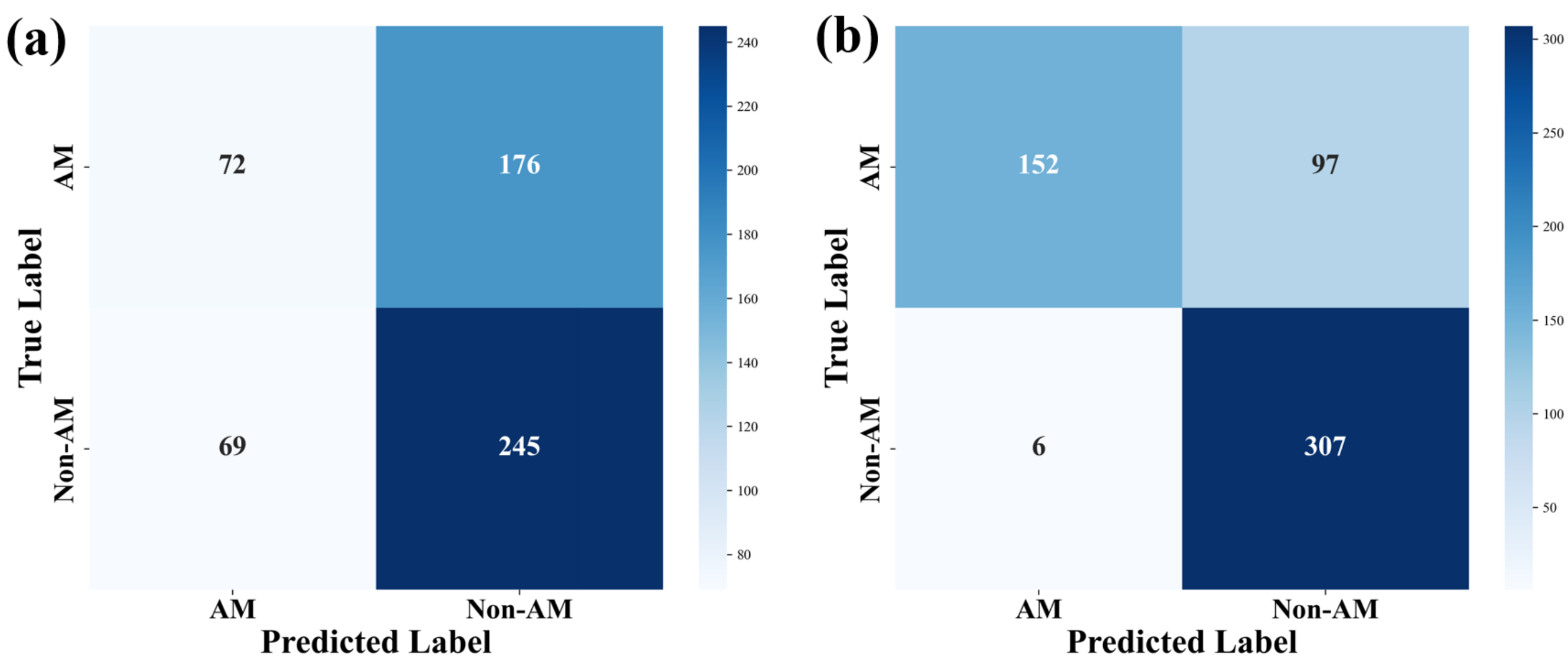

FIG. S2. a) Confusion matrix for the test set, using the model trained on the PD dataset, achieving an accuracy of 56 %. b) Same as panel a but using the model trained on the CD dataset, showing an accuracy of 82 %. Note that the model tends to classify most samples as non-AM.

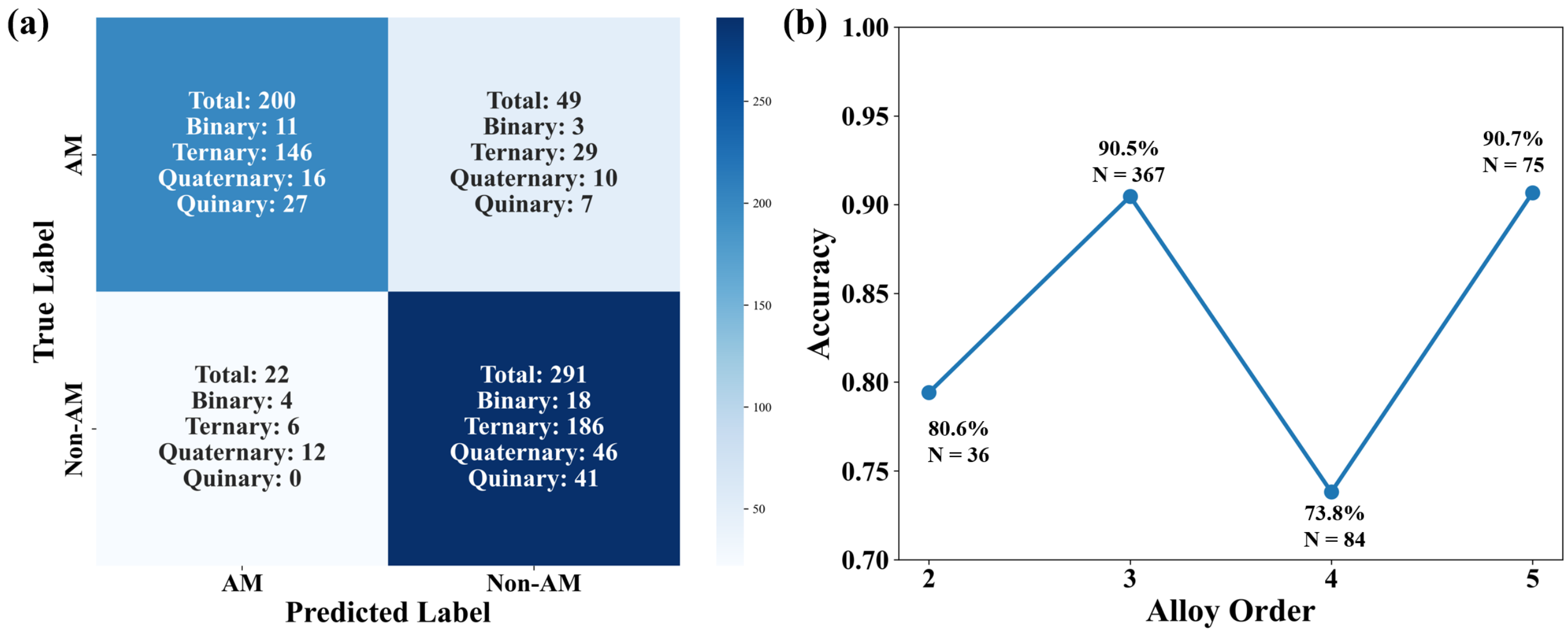

FIG. S3. Performance of the model trained on the EDs dataset by alloy order: a) Confusion matrix and b) Prediction accuracy for different alloy orders, N indicates the number of samples in each alloy-order category.

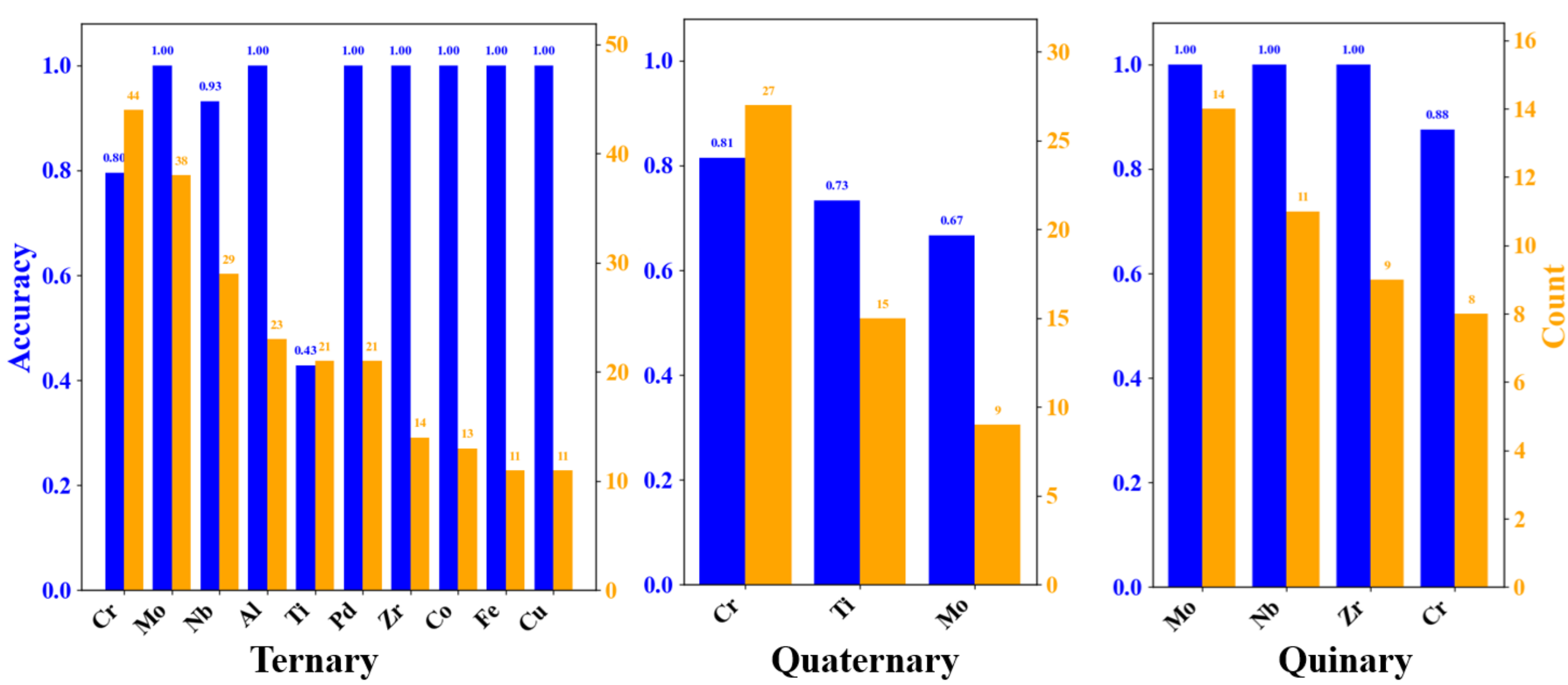

FIG. S4. Accuracy and sample counts by principal-element group for different alloy orders. The binary alloy results are not shown because all principal-element groups contained fewer than 5 samples. For ternary alloys, the principal element was defined as an element with a concentration of at least 50%, whereas for quaternary and quinary alloys, an element with a concentration of at least 40% could be regarded as the main element. In our training set, 269 samples contain Mo, of which 45 are Mo-dominant; 365 samples contain Nb, of which 90 are Nb-dominant; 416 samples contain Cr, of which 25 are Cr-dominant; and 1,232 samples contain Zr, of which 460 are Zr-dominant. It can be seen that the accuracy is quite dependent on the main element of the alloy

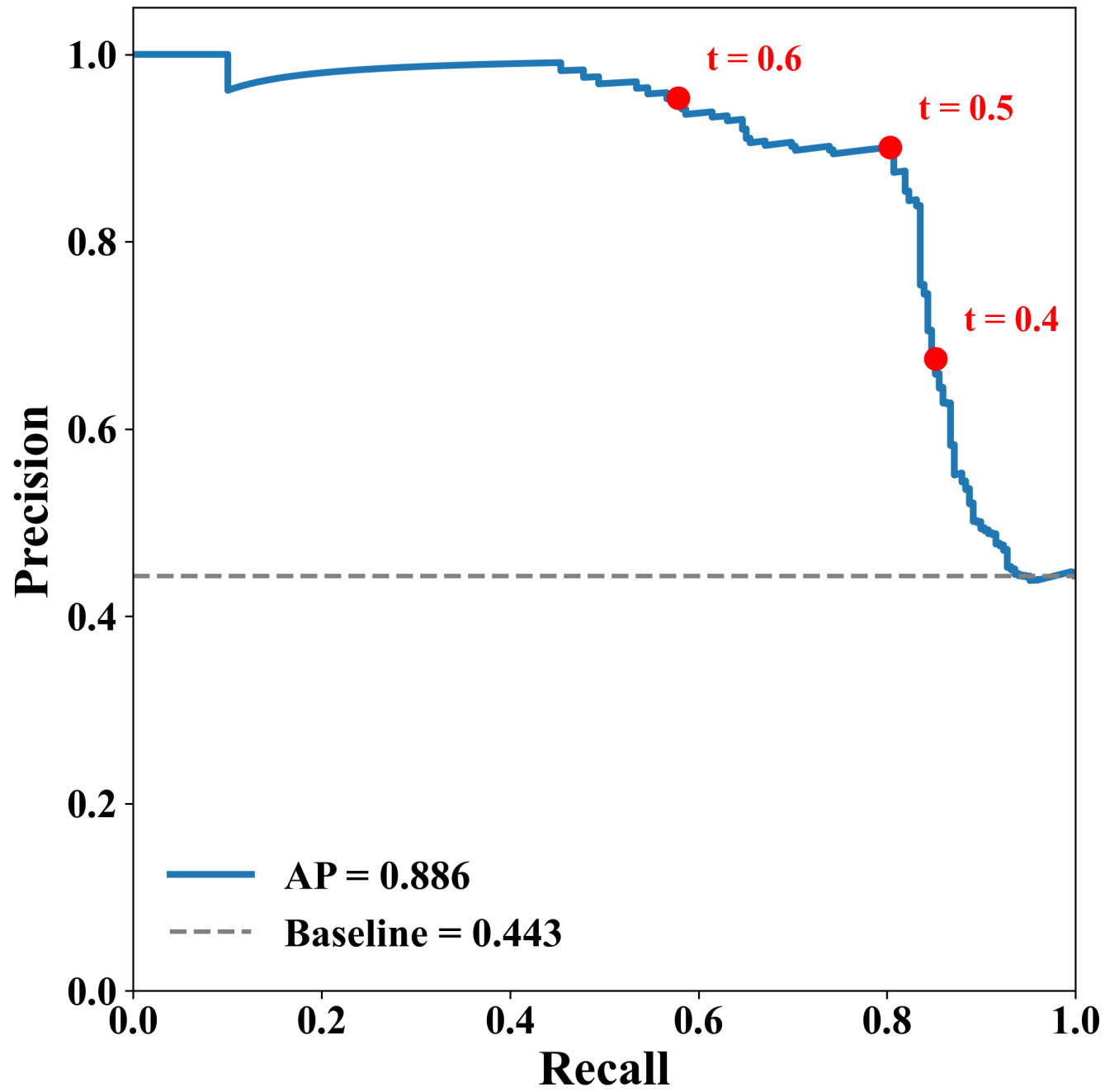

FIG. S5. Precision recall curve of the model trained with EDs, with AM alloys treated as the positive class. The average precision (AP) is 0.886. The red markers indicate decision thresholds (t) of 0.4, 0.5, and 0.6. At t = 0.6, the model achieves very high precision but identifies only about 60% of the amorphous alloys, indicating a conservative decision criterion. Reducing the threshold to t = 0.4 increases the recall to approximately 85%, at the expense of a decrease in precision to about 67% due to more false positive predictions. The threshold of t = 0.5 provides a more balanced result between precision and recall.

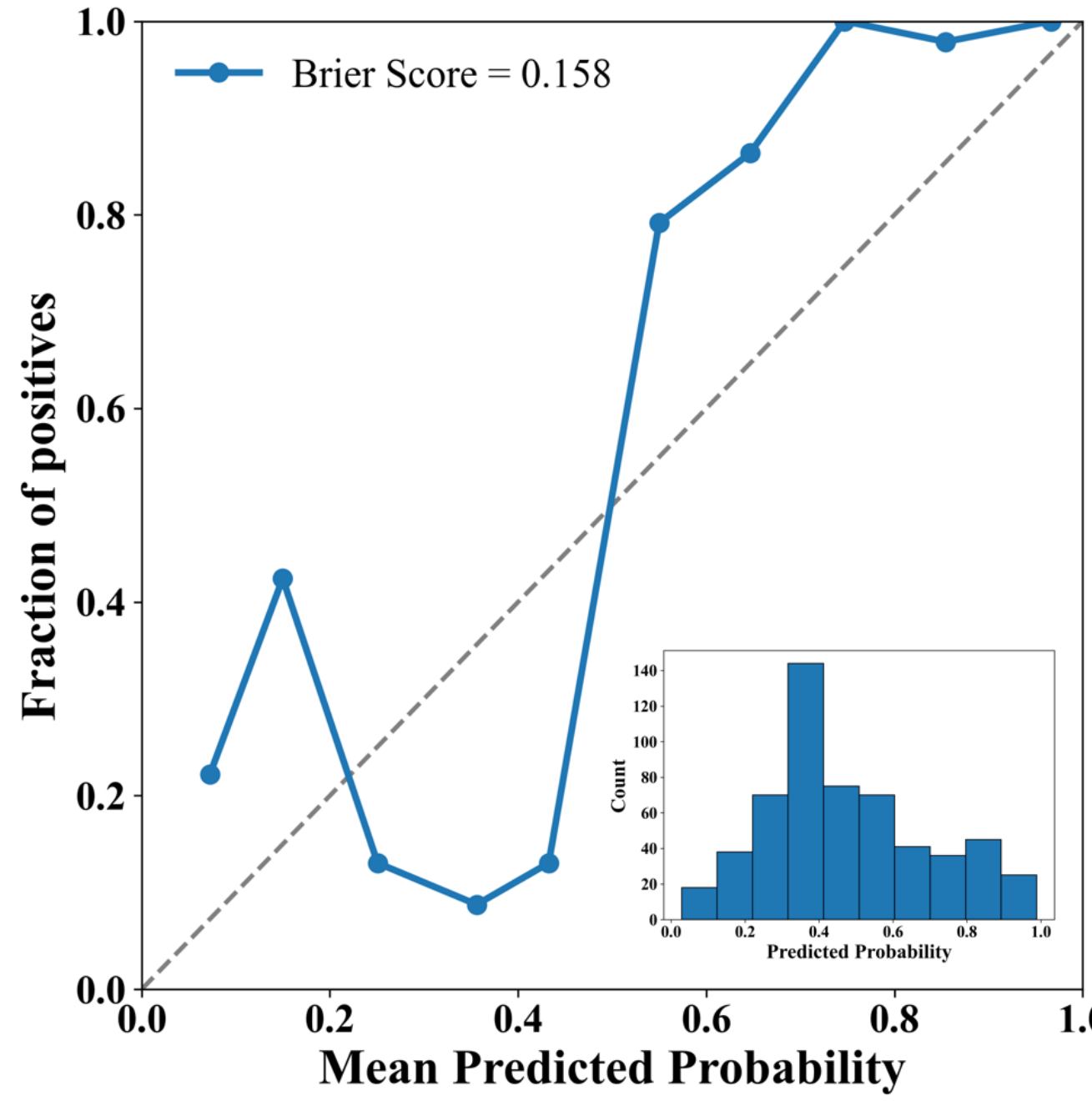

FIG. S6. Calibration curve of the model trained with EDs, with AM alloys treated as the positive class. The calibration curve was constructed by dividing the predicted probabilities into ten equally spaced bins. Each point represents the mean predicted probability and the corresponding observed fraction of AM compositions within the bin. The inset histogram shows the distribution of predicted probabilities across the ten bins used to construct the calibration curve. The calibration curve exhibits a S-shaped trend, indicating that the model tends to overestimate the probability of AM formation at low P_{glass} values and underestimate it at high P_{glass} values. The corresponding Brier score is 0.158. Overall, the predicted probabilities remain broadly consistent with the observed positive frequencies despite the systematic deviations. Since candidate compositions are typically ranked according to P_{glass} , the observed underestimation in the high-probability region is unlikely to affect the prioritization of promising glass forming compositions.

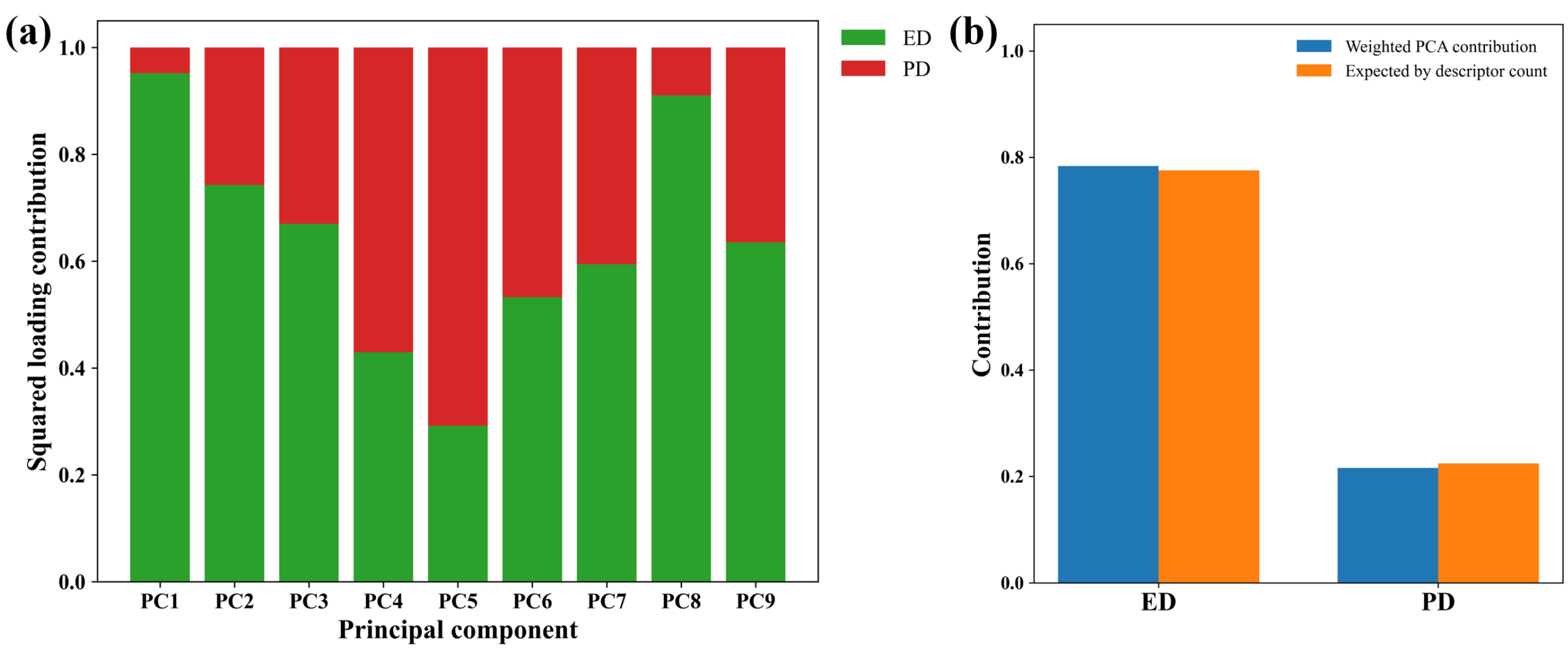

FIG. S7. a) PCA loading contribution of EDs and PDs in the combined descriptor space. green bars represent EDs, and red bars represent PDs. For each principal component, the group contribution of EDs and PDs is calculated as the sum of squared descriptor loading coefficients belonging to that group. b) Explained variance weighted group contribution of EDs and PDs compared with the descriptor count based expected contribution. This comparison accounts for the different numbers of ED and PD descriptors. PDs contribute to the PCA space, especially in PC4 and PC5, and their total weighted contribution is close to the expected value of the number of descriptors. As shown in Fig. 2a in the main text, the first three principal components which are mainly dominated by EDs already explains about 50% of the descriptor variance. Adding PDs introduces additional variance directions into the model, but these directions may not necessarily be helpful for the prediction.

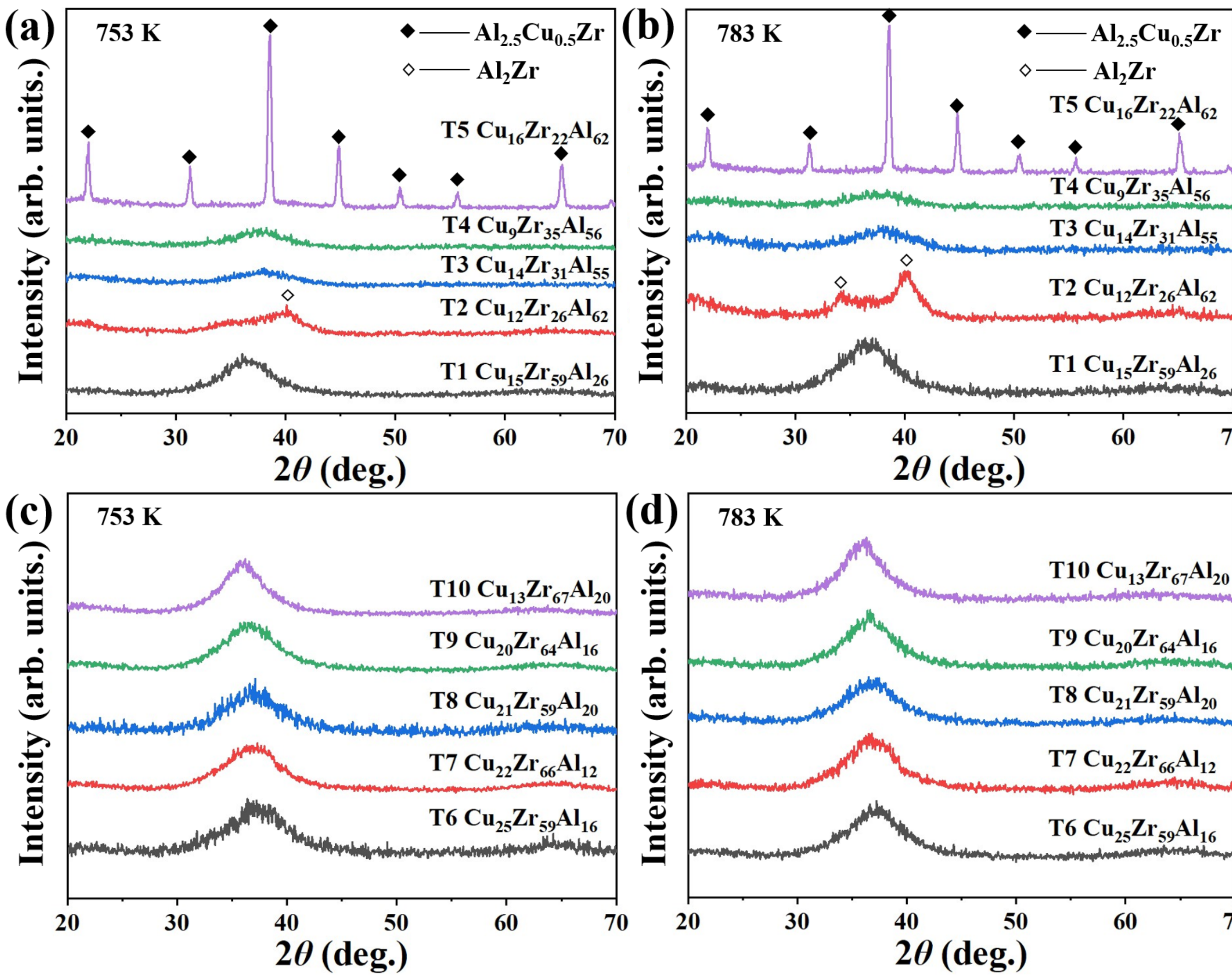

FIG. S8. GIXRD patterns after annealing the samples T1-T5 up to a) 753 K and b) 783 K, using 1 h annealing with 30 K temperature increments. Same results also for the samples T6-T10 for c) 753 K and d) 783 K, using the same process. The peaks in the figures have been identified with the corresponding alloys shown in the legend.

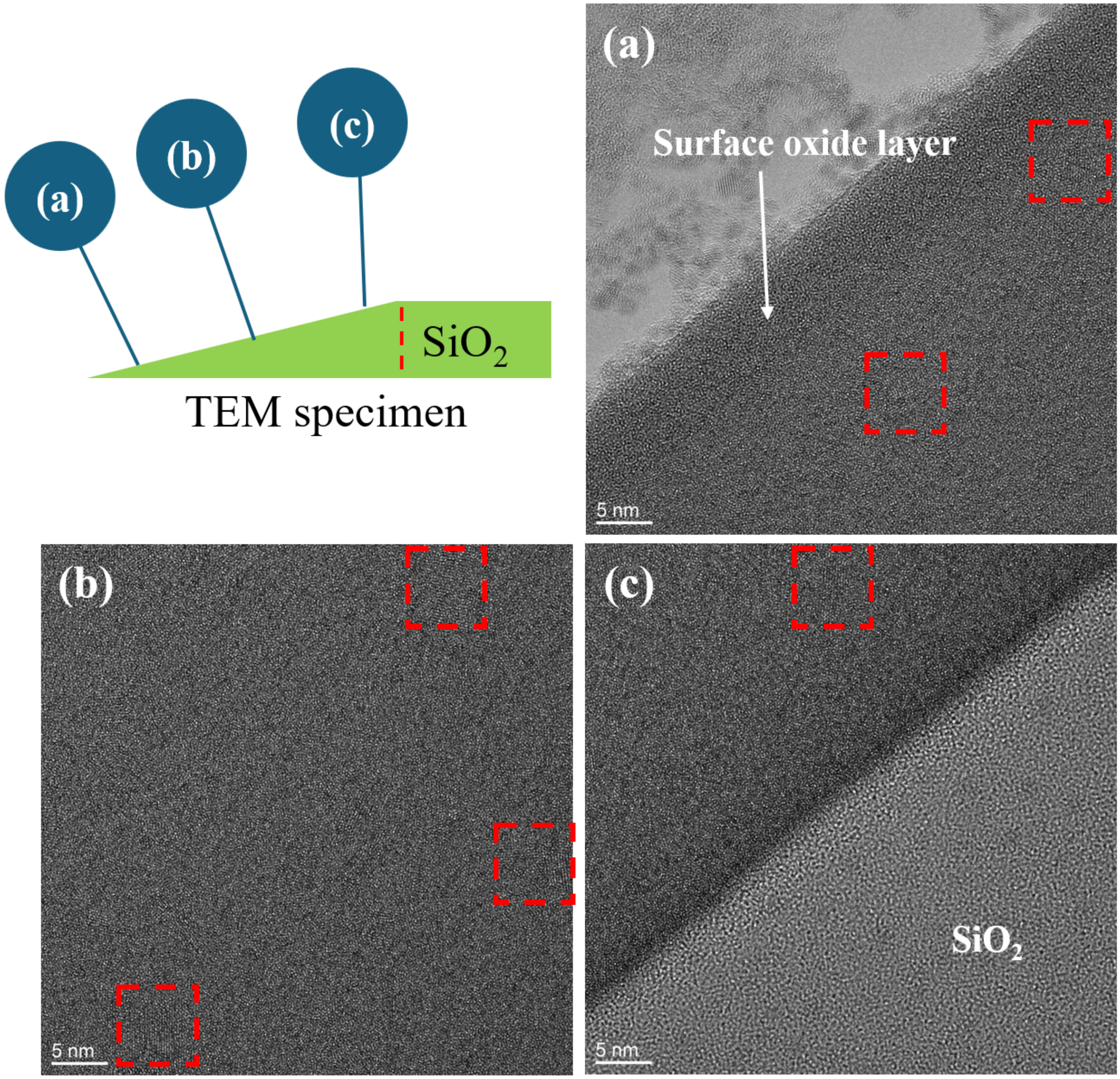

FIG. S9. As shown in the schematic diagram, the HRTEM of the T4 ($Cu_9Zr_{35}Al_{56}$) is shown at a) surface; b) inside and c) interface.

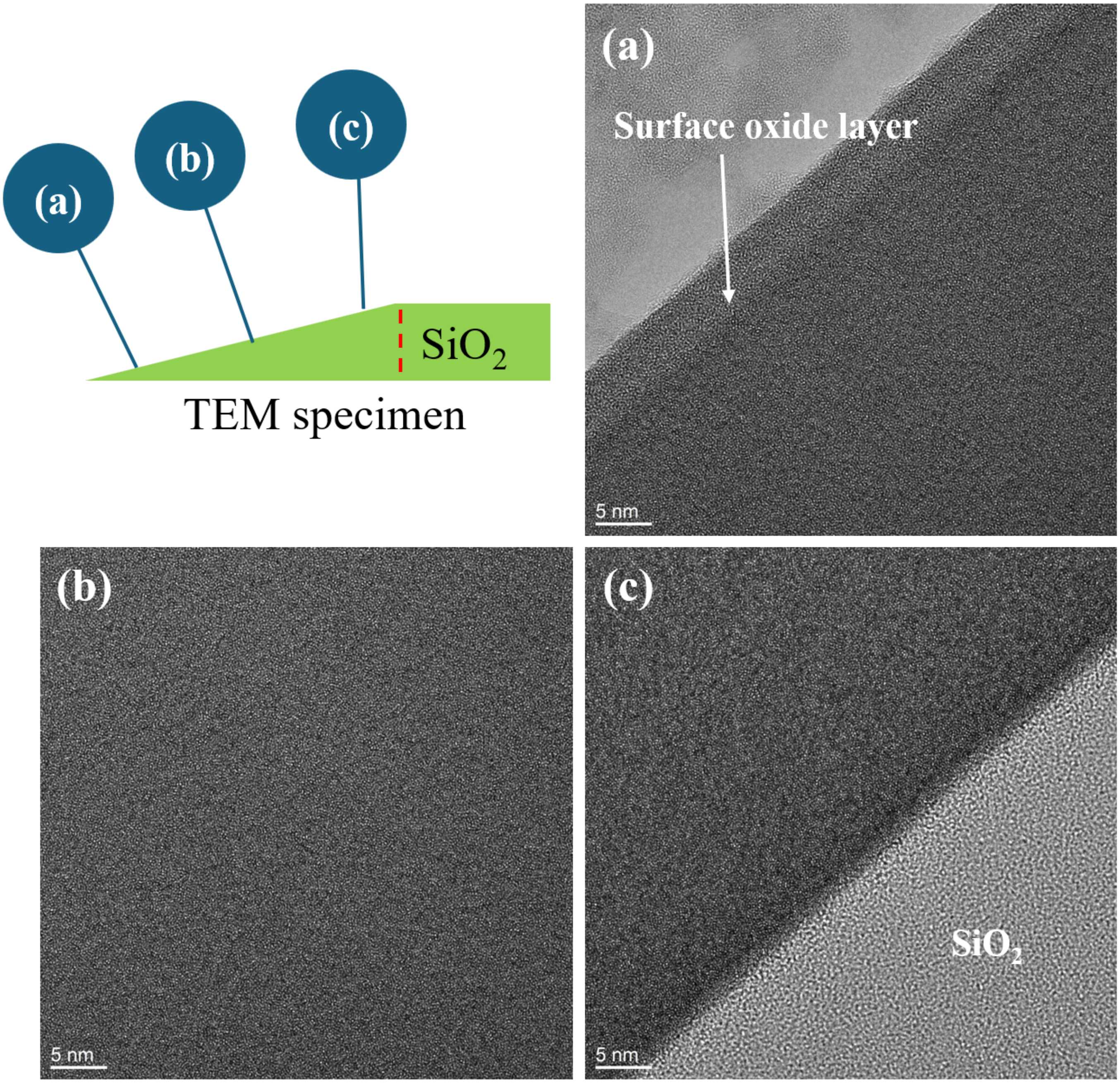

FIG. S10. As shown in the schematic diagram, the HRTEM of the T8 ($Cu_{21}Zr_{59}Al_{20}$) is shown at a) surface; b) inside and c) interface.
